# Digital microfluidic immunocytochemistry in single cells

**DOI:** 10.1038/ncomms8513

**Published:** 2015-06-24

**Authors:** Alphonsus H. C. Ng, M. Dean Chamberlain, Haozhong Situ, Victor Lee, Aaron R. Wheeler

**Affiliations:** 1 Institute of Biomaterials and Biomedical Engineering, University of Toronto, 164 College Street, Toronto, Ontario M5S 3G9, Canada; 2 Donnelly Centre for Cellular and Biomolecular Research, 160 College Street, Toronto, Ontario M5S 3E1, Canada; 3 Department of Chemical Engineering and Applied Chemistry, 200 College Street, Toronto, Ontario M5S 3E5, Canada; 4 Department of Chemistry, University of Toronto, 80 Saint George Street, Toronto, Ontario M5S 3H6, Canada

## Abstract

We report a new technique called Digital microfluidic Immunocytochemistry in Single Cells (DISC). DISC automates protocols for cell culture, stimulation and immunocytochemistry, enabling the interrogation of protein phosphorylation on pulsing with stimulus for as little as 3 s. DISC was used to probe the phosphorylation states of platelet-derived growth factor receptor (PDGFR) and the downstream signalling protein, Akt, to evaluate concentration- and time-dependent effects of stimulation. The high time resolution of the technique allowed for surprising new observations—for example, a 10 s pulse stimulus of a low concentration of PDGF is sufficient to cause >30% of adherent fibroblasts to commit to Akt activation. With the ability to quantitatively probe signalling events with high time resolution at the single-cell level, we propose that DISC may be an important new technique for a wide range of applications, especially for screening signalling responses of a heterogeneous cell population.

## Abstract (web summary)

The ability to measure signalling responses in single cells following short pulses of stimulus would shed insight into temporal thresholds for cell activation. Here the authors introduce a microfluidic platform that allows downstream phosphorylation cascades to be observed following as little as one second of stimulus exposure.

## Graphical abstract

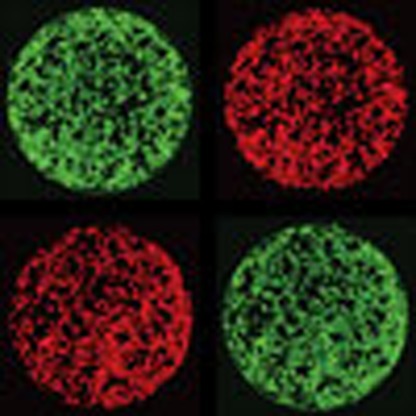

Elucidating the mechanisms that regulate cell function and fate requires the measurement of signalling events in response to perturbation^1^,^2^. These mechanisms can be difficult to study as they involve networks of diverse biochemical reactions that occur at a range of timescales. For example, early signalling events such as cell surface-receptor phosphorylation occur within seconds to minutes after stimulus^3^,^4^, while the subsequent phosphorylation of intracellular signalling molecules and signal transduction to the nucleus occurs in minutes to hours. It is important to investigate such effects in detail, as a given signalling ligand or molecule can lead to diverse cellular responses depending on the frequency, concentration and duration of the stimulus^5^,^6^,^7^,^8^. Finally, most of what is known about cell signalling has been gleaned from evaluating the average responses from large populations of cells (using traditional methods such as western blotting or newer methods involving mass spectrometry^3^); however, each individual cell can encode and decode important information differently^9^,^10^. Thus, there is a great need for tools capable of dissecting the mechanisms and dynamics of single-cell signalling with high temporal resolution.

There are a number of methods that have been adapted to evaluate single-cell phosphorylation dynamics, including mass spectrometry and flow cytometry, which offer high sensitivity and multi-parameter analysis^11^,^12^. Despite these outstanding features, these techniques suffer from two key limitations. First, they require a relatively large number of cells in a disaggregated, single-cell suspension. Thus, adherent cells or tissues must be dissociated, which can disrupt native cell–cell and cell–matrix connections and cause unintended perturbation of cell signalling. Second, existing approaches rely on manual liquid handling for cell culture and sample preparation, which typically allow for reliable time resolution in the scale of minutes. Thus, these approaches do not have the temporal resolution required for the perturbation and analysis of fast signalling events like receptor phosphorylation^13^.

To analyse signalling kinetics in the order of seconds, several platforms have been developed relying on flow-based microfluidics and fluorescent microscopy^14^,^15^,^16^,^17^,^18^,^19^,^20^,^21^,^22^,^23^,^24^. The development and maturation of microdevices^25^ and optical techniques^26^ has been a boon for the study of signalling dynamics in single cells, leading, for example, to seminal findings regarding yeast signalling pathways^16^,^18^,^19^. Microfluidic systems enable the automated delivery of chemical stimulant to cells, and the resulting cellular responses can be imaged in real-time using green fluorescent protein reporters^15^,^16^,^17^,^18^,^19^,^20^, fluorogenic calcium chelators^21^ or fluorescence resonance energy transfer^22^ via live-cell microscopy. For tracking post-transcriptional modifications such as phosphorylation, antibody-based techniques are needed to probe the modified site^12^. Recently, these techniques have been applied to evaluate platelet-derived growth factor receptor (PDGFR) and insulin growth factor receptor to evaluate stimulus-dependent phosphorylation dynamics of Akt^27^. These methods offer an exciting new window into cell signalling, but there are at least two limitations to flow-based microfluidic measurements. First, to achieve fast switching of chemical stimulant (allowing for fine time resolution), high flow rates are required^14^, which (when combined with small channel dimensions) result in levels of shear stress (>1 dyn cm^−2^) that have been shown to cause unwanted flow-mediated signalling^28^,^29^. Second, the serial nature of flow-based microfluidics typically requires that systems incorporate integrated PDMS-based valves to prevent cross-talk between cell culture chambers^30^. Such devices can be tricky to set-up and operate, and great care must be taken to enable cell attachment, prevent adsorption and absorption of biomolecules onto and into the PDMS^31^, and minimize substrate toxicity^32^.

To address the challenges described above, we report here a new droplet-based method called Digital microfluidic Immunocytochemistry in Single Cells (DISC), which can automate all of the steps required to analyse signalling events in adherent single cells *in situ*. Like flow-based microfluidic techniques, DISC enables rapid changes in stimulant concentration coupled with integrated analysis, but DISC is unique in the ability to implement such studies without high levels of shear stress or moving parts. Further, DISC generates single-cell population data that are similar to flow cytometry but in an automated manner with *in situ* adherent cells.

In this work we report the application of DISC to evaluate the well characterized^33^ phosphoinositide 3-kinase (PI3K)/Akt signalling pathway downstream of PDGF stimulation of PDGFR. The results recapitulate what is known about the early stages of the pathway, but the high time resolution of the technique allows for surprising new observations—for example, a 10 s pulse stimulus of low-concentration PDGF is sufficient to cause >30% of adherent fibroblasts to commit to Akt activation. These results are the first of their kind and serve as examples of what may be possible in the future—we propose that DISC may be an important new technique for probing temporal dynamics of signalling in heterogeneous cell populations at a single-cell level for a wide range of applications.

## Results

### Digital microfluidic Immunocytochemistry in Single Cells

**Figure 1:**
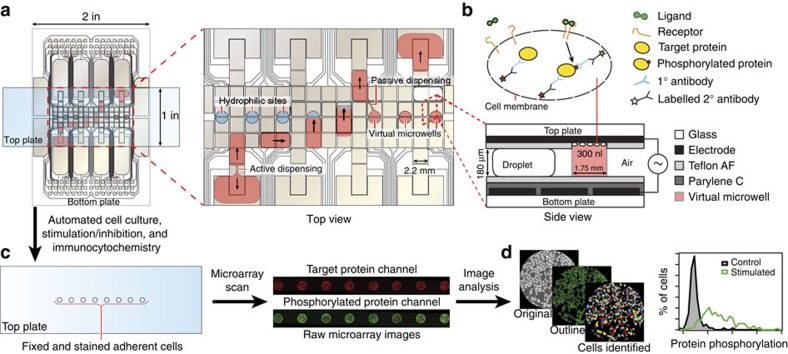
Digital microfluidic Immunocytochemistry in Single Cells (DISC). (**a**) Top-view schematic of a digital microfluidic device used for cell culture, stimulation and immunocytochemistry. The expanded view illustrates the two primary fluid-handling operations, active and passive dispensing, required for metering and delivering reagents to cells cultured in virtual microwells. (**b**) Side-view schematic showing adherent cells cultured on the patterned top plate in a virtual microwell. The cells are sequentially treated with ligand and then probed for protein phosphorylation by immunocytochemistry. (**c**) Cartoon and microarray image illustrating how the top plate (a 3 by 1 inch slide bearing fixed and stained cells) is transferred from the device to a microarray scanner for laser scanning cytometry. (**d**) Images and data illustrating how the scans are processed by CellProfiler (open-source cell image analysis software) to identify individual cells and extract quantitative data. For each cell, the fluorescent intensity from the phosphorylated protein is normalized to the fluorescent intensity from the corresponding whole protein (phosphorylated+non-phosphorylated) to account for differences in expression level.

DISC was developed to address the limitations in current techniques used to evaluate cell signalling. DISC relies on digital microfluidics (DMF) to automate fluid handling in a device comprising two parallel plates that are separated by spacers (Figs. 1a,b). The bottom plate has an array of electrodes that can be used to manipulate droplets to seed and culture adherent cells, deliver stimuli and carry out immunocytochemistry. The top plate contains eight hydrophilic sites for adherent cell culture; when droplets of cell suspension are passed across the hydrophilic sites, virtual microwells (VMs) are formed by a phenomenon known as ‘passive dispensing' (Supplementary Fig. 1 and Supplementary Movie 1)^34^. Using passive dispensing, cells adhered in the VMs are stimulated, fixed and labelled with antibodies for phosphorylated proteins of interest (Fig. 1b, Supplementary Fig. 2 and Supplementary Movie 2). The top plate is then removed from the device and analysed by a microarray scanner (Fig. 1c). The images generated are analysed using CellProfiler to calculate the ratio of each phosphorylated (target) protein to total target protein in each cell (Fig. 1d and Supplementary Fig. 3). In addition, physical and morphological data can be extracted for each individual cell.

**Figure 2:**
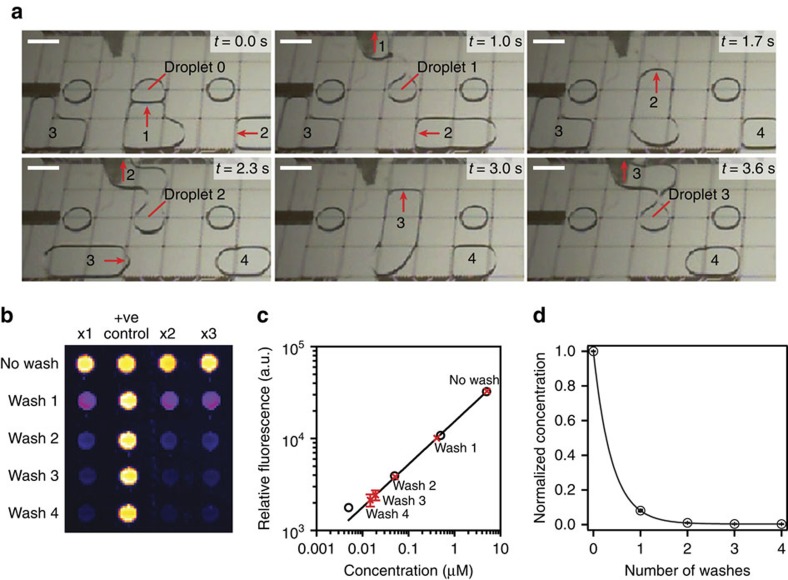
Speed and efficiency of passive dispensing. (**a**) Time-stamped video sequence from Supplementary Movie 3 depicting passive dispensing of three successive 1.8 μl droplets to a virtual microwell—∼1 s is required between two reagent exchanges. Scale bars, 2 mm. (**b**) Fluorescent scan of virtual microwells (VMs) initially filled with 5 μM fluosceinamine, washed 0, 1, 2, 3 or 4 times (*y*-axis) with 1.8 μl droplet(s) of wash buffer (3 technical replicates on the *x*-axis: x1, x2, x3). (**c**) Plot of standard curve (open black circles) generated from virtual microwells filled with 5, 0.5, 0.05 or 0.005 μM fluosceinamine overlayed with interpolated data points from VMs washed with 0, 1, 2, 3 or 4 droplets (red crosses). Error bars are ±1 s.d. from x1–x3. (**d**) Plot showing the efficacy of passive dispensing for removing 5 μM fluoresceinamine from virtual microwells as a function of the number of wash droplets. Error bars are ±1 s.d. from x1–x3.

The DISC platform has the ability to perturb and analyse cells with high temporal resolution, an important property that is lacking in most analysis systems^10^. Specifically, it was found that ∼1 s elapses between reagent exchanges. As shown in Fig. 2a and Supplementary Movie 3, displacement of droplet 0 by droplet 1 requires 1 s; successive end-to-end replacement of reagents (droplets 1 and 2 or droplets 2 and 3) requires ∼1.3 s. This fast switching enables cells to be stimulated (with ligand in droplet 1) for ∼1 s before fixing (with formaldehyde in droplet 2). A related capability is the ability to perform very efficient reagent exchange. Using fluorescent tracers, it was found that one reagent exchange (with a single droplet) displaces ∼93% of the original solution in the VM, while two reagent exchange displaces >99% (Fig. 2b–d).

To probe the analytical capabilities of the new method, cells were stained and evaluated on-chip for various seeding densities of fibroblasts ranging from 10^3^ to 10^6^ cells per ml (Supplementary Fig. 4). The results demonstrated that 10^5^ to 10^6^ cells per ml are sufficient for analysis, yielding ∼150–300 cells in each VM (2.4 mm^2^). As shown in Supplementary Fig. 5, the signal (measured by labelling with primary and secondary antibody) was significantly (at least 5 × ) higher than background signal (measured by labelling with secondary antibody only) when averaged across the entire VM. (As described below, this sensitivity improves further when applied to single-cell analysis since the background noise outside of the cells is ignored.) Compared with 96-well-plate assays, the DISC method uses 60–150 × fewer cells (15,000 cells per well versus 150–300 cells per VM) and enables significant reductions in working volume 50 μl per well versus 400 nl per VM).

### DISC validation

**Figure 3:**
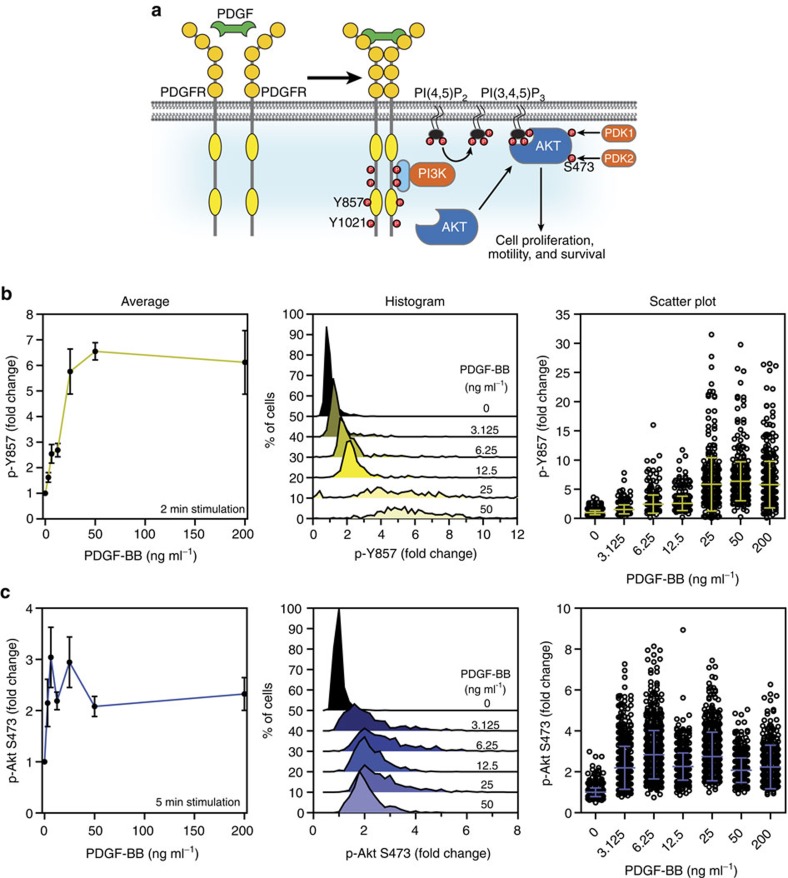
Analysis of the PDGFR/PI3K/Akt pathway in NIH-3T3 fibroblasts by PDGF-BB stimulation. (**a**) Cartoon illustrating signal transduction in PDGFR/PI3K/Akt pathway. (**b**) Dose–response plots of PDGFR Y857 2 min after stimulation, and (**c**) Akt S473 phosphorylation 5 min after stimulation. Data are expressed as fold changes relative to the response of cells exposed to a droplet containing only serum-free DMEM (that is, no stimulation). The ‘Average' plots (left) are the average levels ±s.e.m. from all of the cells in three to four virtual microwells performed on different days. Representative ‘Histograms' (middle) and single cell ‘Scatter plots' (right) are shown with population mean±1 s.d. (representing a total of 383–1,288 cells per condition). Histogram distributions were offset vertically for comparison.

The well-characterized^33^ PDGFR/PI3K/Akt pathway was chosen as a model system to validate DISC for investigating cell signalling. As shown in Fig. 3a, when PDGF binds to PDGFR, the latter auto-phosphorylates, creating binding sites for PI3K, which catalyses the transformation of lipid PI(4,5)P to PI(3,4,5)P. The PI(3,4,5)P then recruits Akt to the plasma membrane, where it becomes phosphorylated, which leads to downstream cascades involved in proliferation, differentiation, metabolism and migration^35^. Two PDGFR tyrosine phosphorylation sites (Y857 and Y1021) and one Akt serine phosphorylation site (S473) were probed in NIH-3T3 fibroblasts after stimulation by delivery of droplets containing various concentrations of PDGF.

Cell responses were first probed at the bulk or average level (with no differentiation between individual cells). As expected, there was a dose-dependent increase in receptor phosphorylation with stimulation using increasing concentrations of PDGF (Fig. 3b, left panel and Supplementary Fig. 6, left panel). As shown, the phosphorylation of PDGFR tyrosine 857 and 1021 sites starts at a PDGF concentration of 3.125 ng ml^−1^ and increases with increasing concentrations of PDGF until 50 ng ml^−1^ of PDGF where it plateaus. Using this dose–response data (Supplementary Fig. 7), the Hill coefficients were calculated to be 1.73±0.33 and 2.13±0.44 and the half-maximum activation concentrations were calculated to be 15.89±1.86 ng ml^−1^ and 18.33±1.94 ng ml^−1^ for Tyr-857 and Tyr-1021, respectively, meaning that the receptor experiences cooperative ligand binding (the ±values represent the s.e. of parameters in the non-linear regression of 21 points). These data are consistent with previous reports of Hill coefficient and half-maximum activation concentration^36^. Again probing cell behaviour at the bulk level, the activation of PDGFR was observed to lead to downstream activation of Akt (Fig. 3c, left panel). Interestingly, the downstream response is amplified—for example, when stimulated with 3.125 ng ml^−1^ PDGF there is a ∼22% phosphorylation of the receptor, while at the same stimulation concentration the Akt phosphorylation is >70%. This is consistent with the findings of Park *et al.*^36^, who observed that only 10% of maximum receptor phosphorylation is needed to achieve a 50% activation of Akt. Overall, the ‘average' results (Fig. 3b,c, left panels and Supplementary Fig. 6, left panel) are consistent with previous observations, suggesting that the DISC platform does not alter or disrupt the expected behaviour of PDGFR signalling pathways.

Analysis of average cellular response, similar to that described above, can be performed using established methods such as western blots. However, DISC can also evaluate heterogeneous signalling response of cell populations at the single-cell level. For example, the phosphorylation states in individual cells can be presented as histograms (Fig. 3b,c, middle panel and Supplementary Fig. 6, middle panel), which highlights the distribution of the observed responses and enables straightforward comparison between populations treated with different conditions. At ligand concentration of 0, all of the cells exhibited basal level of receptor phosphorylation, as illustrated in the tall, narrow histograms at fold change of 1. Stimulation with 3.125 ng ml^−1^ of PDGF triggered phosphorylation of the receptor, shown by the rightward shift in the histograms, but the population remains relatively homogenous as indicated by the narrow histograms. When PDGF concentration was increased to 50 ng ml^−1^, there was an increase in the distribution of receptor phosphorylation states by the individual cells as shown by the increasing width of the histograms. Alternatively, the phosphorylation states of individual cells can be represented as scatter plots (Figs 3b,c, right panel and Supplementary Fig. 6, right panel), which is useful for identifying outlier members of the population such as hyperactive responders. This information about how individual cells respond to PDGF stimulation is lost with traditional methods.

The data described above suggest that there is little (if any) bias inherent in DISC methodology. But the novelty of the technique led us to test explicitly whether the shear stress (∼0.5 dyn cm^−2^ as per Supplementary Note 1) or other effects of droplet movement have any effect on the signalling events described here. To test this hypothesis, Akt phosphorylation was observed under an ‘extreme' condition in which ligand-free droplets were continuously moved back-and-forth across cells 75 times over 5 min. As shown in Supplementary Fig. 8, the response for cells treated in this manner is not significantly different (*P*=0.7188, two-tailed Student's *t*-test) from undisturbed cells. These results provide further confidence that DISC methodology does not alter the expected behaviour of the signalling pathway.

### DISC for cell heterogeneity

After validating DISC as a reliable method for analysing the PDGFR/PI3K/Akt signalling pathway, we turned to a related application to probe the capacity for DISC to enable a deep-dive analysis of single-cell behaviour. Two breast cancer cells lines (MCF-7 and MDA-MB-231) were chosen in recognition of the growing importance placed on understanding the relationship between cancer cell heterogeneity (particularly in the PDGFR system^37^) and chemotherapeutic efficacy. As shown in Supplementary Fig. 9a, on stimulation with PDGF ligand, MCF-7 and MDA-MB-231 cells exhibit similar bulk response between 0 to 5 min of stimulation, after which MDA-MB-231 cells have significantly greater levels of Akt activation. When viewed at the single-cell level (Supplementary Fig. 9b,c) it is apparent that both cell types are quite heterogeneous, with a significant number of hyperactive responders to stimulation. These hyper-responders can be identified using statistical tests (Supplementary Fig. 10 and Supplementary Fig. 11), which are able to differentiate between the two cell lines within 3 min of stimulation, several minutes before the differences in bulk population activation can be observed.

**Figure 4:**
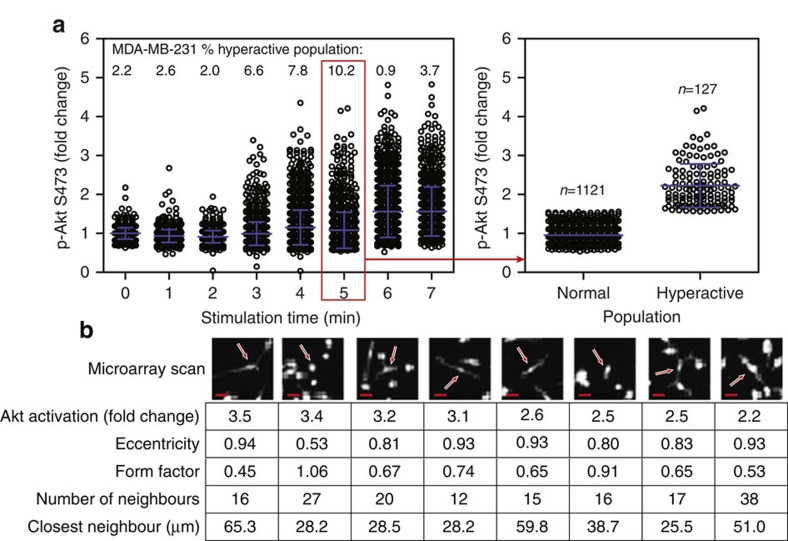
Single-cell Akt S473 phosphorylation in MDA-MB-231 cells. (**a**) Left: single-cell scatter plots of Akt activation after 0–7 min stimulation by 50 ng ml^−1^ of PDGF-BB. Data are shown as population mean±1 s.d. (representing a total of 946–2,219 cells per condition), with the percentage of hyperactive responders indicated above each time point. The most hyperactive responders (red box) were observed at 5 min stimulation. Right: differential single-cell scatter plots of Akt activation for the hyperactive and normal populations (population mean±1 s.d. representing 1,121 normal cells and 127 hyperactive cells) 5 min after stimulation. (**b**) Microarray scans of eight representative hyperactive cells observed 5 min after stimulation (red arrows indicate each cell) and table summarizing each cell's Akt phosphorylation level, shape parameters and neighbour analysis. Scale bars, 25 μm.

Akt phosphorylation results for MDA-MB-231 cells at different times of stimulation are shown in Fig. 4a, left. The condition with the highest numbers of hyperactive cells (5 min stimulation, Fig. 4a, right) was selected for detailed analysis of cellular properties, including size (area and perimeter), shape (eccentricity and form factor) and neighbour proximity (quantity of close neighbours and distance to closest neighbour). Representative microarray images of hyperactive cells as well as tables of their properties are shown in Fig. 4b. When compared with normal cells, hyperactive cells were found to have statistically indistinguishable size (Supplementary Fig. 12). In contrast, hyperactive cells were found to have statistically significant differences in shape (Supplementary Fig. 13)—that is, hyperactive cells are more eccentric and have reduced form factors relative to normal cells—all factors that correlate with an elongated morphology^38^. Likewise, hyperactive cells were found to have more and closer neighbours relative to normal cells (Supplementary Fig. 14), suggesting that paracrine signalling has some effect on hyperactivity^39^,^40^. Given this observation of cell-position effects (relative to other cells), as a control, the same data were evaluated as a function of cell position in the wells. As shown in Supplementary Fig. 15, cell position within the VM did not have a measurable effect on Akt activation, suggesting that ligand gradients, edge effects or other position-related effects do not play a role in this observation. Obviously, the type of information gleaned from Supplementary Figs 12–14 is only available for methodologies such as DISC that can retain both spatial and shape information for cells under investigation.

### DISC for time-resolved signalling

DISC was developed to study cell signalling processes at high temporal resolution, with stimulation durations lasting as little as 1 s prior to fixing and analysis. This is particularly useful when evaluating fast processes such as receptor phosphorylation. To test this ability, we used DISC to carry out time-resolved receptor phosphorylation analysis of fibroblasts for two types of time-resolved ligand stimulation: (1) single-bolus stimulation, and (2) double-bolus stimulation.

**Figure 5:**
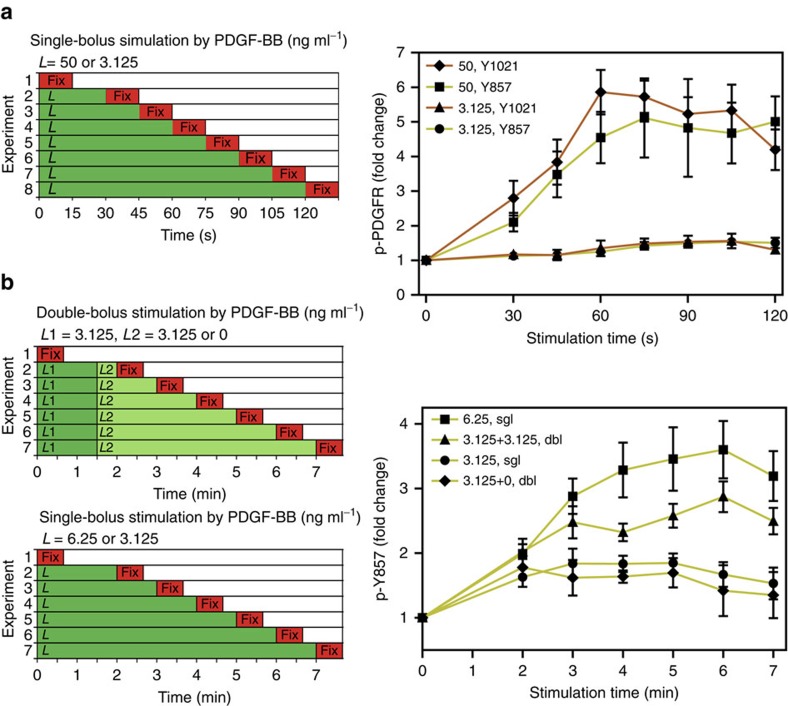
Time-resolved analysis of the PDGFR/PI3K/Akt pathway in NIH-3T3 fibroblasts by PDGF-BB stimulation (stimulation experimental schemes on the left, plots on the right). (**a**) PDGFR Y857 and Y1021 phosphorylation as a function of stimulation time (0–120 s). Cells were exposed to a single bolus of 50 ng ml^−1^ (Y1021=diamonds, Y857=squares) or 3.125 ng ml^−1^ (Y1021=triangles, Y857=circles) of PDGF-BB. (**b**) PDGFR Y857 phosphorylation as a function of stimulation time (0–7 min). Cells were exposed to either a double bolus (dbl) or single bolus (sgl) of PDGF-BB. In dbl stimulation, cells are exposed to two boluses of 3.125 ng ml^−1^ (3.125 at *t*=0 s+3.125 at *t*=90 s (triangles)) or one bolus of 3.125 ng ml^−1^ and one of vehicle (3.125 at *t*=0 s+0 at *t*=90 s (diamonds)). In sgl stimulation, cells were exposed to one bolus of 6.25 ng ml^−1^ at *t*=0 s (squares) or 3.125 ng ml^−1^ at *t*=0 s (circles). Data in the kinetic plots are average fold changes with respect to zero (no stimulation) ±s.e.m. from at least 3–11 virtual microwells performed on different days (representing a total of 391–1,063 cells per condition).

In the first type of test, PDGFR tyrosine phosphorylation was evaluated after stimulating fibroblasts with a single bolus of high (50 ng ml^−1^) or low (3.125 ng ml^−1^) concentrations of PDGF (Fig. 5a). At high PDGF concentration, there is rapid phosphorylation of the receptor to a maximal level at 75 s and 60 s after stimulation for the phosphorylation sites Tyr-857 and Tyr-1021, respectively. This behaviour is similar to that reported in the study by Park *et al.*^36^, but in their work, the earliest time point was 2 min, which obscured the determination of the time of maximal receptor phosphorylation. At low PDGF concentration, there is a low level of receptor phosphorylation at both the phosphorylation sites. This might be caused by signal repression of the receptor phosphorylation by a phosphatase or alternatively it could simply be caused by a low ratio of ligand molecules to receptors. To test this, the receptor was stimulated in the presence or absence of the phosphatase inhibitor, orthovanadate. As shown in Supplementary Fig. 16, the inhibitor did not cause a significant difference (*P*=0.1163, two-way analysis of variance) in the phosphorylation pattern, suggesting that the low level of phosphorylation is caused by a low ligand-to-receptor ratio and not phosphatase activity.

In the second type of test, PDGFR phosphorylation in fibroblasts was evaluated after stimulation of two boluses of PDGF. Initially, all cells were exposed to 3.125 ng ml^−1^ PDGF. After 90 s, some cells received a second bolus of 3.125 ng ml^−1^ ligand while other cells received a bolus of 0 ng ml^−1^ ligand. As a reference, receptor phosphorylation was also evaluated for cells exposed to a single bolus (with no second bolus) of 3.125 or 6.25 ng ml^−1^ PDGF. As shown in Fig. 5b, when cells were addressed with a double-bolus stimulation (3.125+3.125 ng ml^−1^ ligand), the receptor phosphorylation level was higher than that of the control (3.125+0 ng ml^−1^ ligand), but between the levels observed for cells stimulated with a single bolus of 3.125 ng ml^−1^ and a single bolus of 6.25 ng ml^−1^ PDGF. Further, the control double-bolus stimulation (3.125+0 ng ml^−1^ ligand) resulted in a similar receptor phosphorylation level as a single bolus of 3.125 ng ml^−1^ ligand, suggesting that 90 s of exposure to ligand (after which it was washed away) was sufficient for maximal receptor phosphorylation for that concentration.

**Figure 6:**
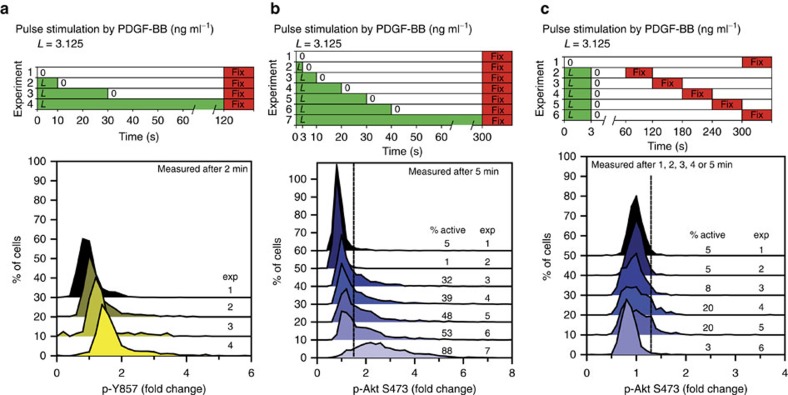
Pulse stimulation of NIH-3T3 fibroblasts by PDGF-BB (experimental stimulation schemes on the top, histograms on the bottom). (**a**) PDGFR Y857 phosphorylation measured 2 min after stimulation (top-to-bottom) with 0 ng ml^−1^ PDGF-BB at *t*=0 s with no wash (exp 1, negative control), 3.125 ng ml^−1^ at *t*=0 s with wash at *t*=10 s (exp 2), 3.125 ng ml^−1^ at *t*=0 s with wash at *t*=30 s (exp 3), 3.125 ng ml^−1^ at *t*=0 s with no wash (exp 4, positive control). (**b**) Akt S473 phosphorylation measured 5 min after stimulation (top-to-bottom) with 0 ng ml^−1^ PDGF-BB at *t*=0 s with no wash (exp 1, negative control), 3.125 ng ml^−1^ at *t*=0 s with wash at *t*=3 s (exp 2), 3.125 ng ml^−1^ at *t*=0 s with wash at *t*=10 s (exp 3), 3.125 ng ml^−1^ at *t*=0 s with wash at *t*=20 s (exp 4), 3.125 ng ml^−1^ at *t*=0 s with wash at *t*=30 s (exp 5), 3.125 ng ml^−1^ at *t*=0 s with wash at *t*=40 s (exp 6) and 3.125 ng ml^−1^ at *t*=0 s with no wash (exp 7, positive control). (**c**) Akt S473 phosphorylation (top-to-bottom) measured 5 min after stimulation with 0 ng ml^−1^ PDGF-BB at *t*=0 s with no wash (exp 1, negative control), or 1 min (exp 2), 2 min (exp 3), 3 min (exp 4), 4 min (exp 5) or 5 min (exp 6) after stimulation with 3.125 ng ml^−1^ PDGF-BB at *t*=0 s with wash at *t*=3 s. The dotted vertical lines in **b**,**c** represent a threshold of Akt activation chosen such that the negative control (exp 1) has >95% of cells inactivated; the numbers under the '% active' headings indicate the percentages of cells observed to be above the threshold for each condition. Histogram distributions were generated from at least 3 different day replicates (representing a total of 332–2,501 cells per condition), and were offset vertically for comparison.

Intrigued by the results above, we turned to faster double-bolus wash-away experiments, evaluating both PDGFR and Akt phosphorylation. In these experiments, cells were initially exposed to a bolus of 3.125 ng ml^−1^ ligand, and then a second bolus of ligand-free media to wash it away after a duration of 3 to 40 s, such that the cells were ‘pulsed' with ligand. For comparison, single-bolus (non-pulse) controls were conducted in which cells were exposed to a single bolus of ligand-free media or 3.125 ng ml^−1^ ligand and not washed away until fixation and analysis. After stimulation, cells were fixed and stained for analysis of PDGFR phosphorylation after 2 min (Fig. 6a) or Akt phosphorylation after 5 min (Fig. 6b). As shown, there was detectable activation of PDGFR and Akt after stimulation with a pulse of only 10 s, a result that has not been reported previously. These activation levels are significant—for example, a 10 s pulse of ligand was enough to commit 32% of cells to activate Akt. In contrast, a 3 s pulse of ligand was not sufficient to commit activation of Akt (and was indistinguishable from the control). We then interrogated Akt phosphorylation at earlier time points after 3 s pulses of ligand (Fig. 6c). Interestingly, a small population of cells (20%) initially exhibited Akt activation at 3 and 4 min after stimulation, but was deactivated when evaluated at 5 min after stimulation. This observation is the first of its kind, but is consistent with the ability of cells to filter out high-frequency fluctuations or noise from the environment through enzymatic phosphorylation/dephosphorylation reactions^41^. These data serve as a demonstration of the unique capability of DISC to evaluate complex stimulation patterns at high time resolution.

## Discussion

The new method reported here (Fig. 1) is powered by DMF, which enables software-reconfigurable, parallel-scale distribution of discrete droplets to adherent cells, allowing for reagent delivery in the order of seconds (Fig. 2). The new method builds from several foundational studies that have established proof-of-principle of the utility of DMF for applications involving cells^42^,^43^,^44^,^45^,^46^,^47^,^48^,^49^. As far as we are aware, DISC is the first DMF-based technique capable of rapid cell stimulus with precise resolution; moreover, DISC is the first robust single-cell analysis workflow, enabling multi-parameter kinetic observations ranging from protein phosphorylation and localization to cell morphology.

One of the primary reasons for using DMF in this work is the low shear stress (∼0.5 dyn cm^−2^, Supplementary Note S1) associated with the technique, that allows weakly adhered cells^46^ and cells grown in hydrogels with low viscoelastic modulus^45^ to be washed with negligible cell loss or damage. Receptor-mediated signalling is particularly sensitive to mechanical stress; indeed, shear stresses as low as 1 dyn cm^−2^ have been shown to activate intracellular Erk signalling^29^. This effect was carefully evaluated in DISC, and cell signalling was not induced even under long durations (up to 5 min) of continuous droplet movement (Supplementary Fig. 8). Furthermore, while stressors other than shear (for example, electrical or thermal effects) were not explicitly evaluated here, previous studies^50^ have reported that DMF actuation has negligible effects on cell phenotype when probed for targeted (quantitative PCR for stress-related genes) and untargeted (complementary DNA microarrays) expression changes. In the experiments reported here, three adherent cell lines (NIH-3T3, MCF-7 and MDA-MB-231) were evaluated; but in principle, DISC should be compatible with any adherent cell type including primary cells^47^.

Despite being new, the DISC method is highly accessible. The devices are straightforward to fabricate (only one mask design, no substrate-bonding and so on) and the electronic control system is readily available as open-access hardware^51^. Because the cells in a DISC device are fixed on a standard 3 by 1 inch glass slide, it is trivial to interface them with off-the-shelf detectors, including the microarray scanner used here. Microarray scans have sufficient sensitivity and resolution^52^ for analysis of fluorescently stained single cells, but as shown in Supplementary Movie 4 DISC is compatible with live-cell imaging and microscopy, and in the future we propose that it would be straightforward to combine DISC with high-content screening to probe sub-cellular effects.

DISC was validated by evaluating the PDGFR/PI3K/Akt pathway in response to PDGF ligand stimulation in fibroblasts. The bulk responses (averaged across populations of cells) mirrored what is known about this pathway from conventional methods (for example, western blots)^33^,^36^. When viewed at the single-cell level, the data provides new insights regarding the heterogeneity of receptor activation, revealing that increasing concentration of ligand results in greater variation of receptor phosphorylation, and thereby broadens the individual cellular responses (Fig. 3b and Supplementary Fig. 6). Interestingly, this increase in variability is less apparent in the distribution of phosphorylation states in Akt (Fig. 3c), suggesting the presence of a regulatory mechanism to prevent overactivation of this important downstream target.

After validation, DISC was applied to an in-depth study of single-cell behaviour in an application known to depend on cell heterogeneity^1^,^8^,^10^,^37^, cancer cell signalling. Akt phosphorylation in two breast cancer cell lines (MDA-MB-231 and MCF-7) with known differences in metastatic potential^53^ (that is, the former exhibit increased proliferation and migration rates and reduced susceptibility to apoptosis agonists relative to the latter) were evaluated using DISC. MDA-MB-231 cells were found to have dramatically different numbers of hyperactive responders to PDGF stimulation (Fig. 4 and Supplementary Fig. 9), suggesting that the presence of hyperactive responders (rather than bulk population) may be related to cancer phenotype. Further, it was found that hyperactive responders are elongated and less spherical relative to non-responders (Supplementary Fig. 13). As shown in Supplementary Fig. 17, this effect is a result of cells changing shape during the course of the experiment (not simply and observation of cells that were elongated *a priori*). More study is merited, but this trend is consistent with the transformation that metastatic cells undergo when preparing for migration via epithelial–mesenchymal transition^54^.

A final series of experiments was developed to highlight the most unique aspect of DISC—evaluation of fast ligand–receptor kinetics. Somewhat surprisingly, it was observed that 3.125 ng ml^−1^ of PDGF ligand requires <90 s to achieve maximal receptor phosphorylation potential (Fig. 5b). Furthermore, pulse stimulation with as little as 10 s is sufficient to commit >30% of cells to activate Akt downstream (Fig. 6b)—these kinetics are far faster than any previous study has reported, suggesting that the PDGFR on some cells may be primed to respond faster than other cells in the same population. A greater understanding of this mechanism could be important for drug design and cancer research. The results described here are the first of their kind and serve as examples of what may be possible in the future. We propose that DISC may be an important new technique for probing temporal dynamics of signalling in a heterogeneous population at the single-cell level for a wide range of applications.

## Methods

### Reagents and materials

Unless otherwise specified, reagents were purchased from Sigma-Aldrich (Oakville, ON) (including human recombinant PDGF-BB). Deionized (DI) water had a resistivity of 18 MΩ cm at 25 °C. Dulbecco's Modified Eagle's Medium (DMEM), 10% Pluronic F68 solution and Dulbecco's Phosphate-Buffered Saline (DPBS) with no calcium and magnesium (−/−) were purchased from Life Technologies (Carlsbad, CA). Antibodies were purchased from Santa Cruz Biotechnology (SCB) (Dallas, TX) and Cell Signaling Technologies (CST) (Danvers, MA). Clean room reagents and supplies included S1811 photoresist and MF-321 photoresist developer from Rohm and Haas (Marlborough, MA), CR-4 chromium etchant from Cyantek (Fremont, CA), AZ-300T photoresist stripper from AZ Electronic Materials (Somerville, NJ), Teflon-AF from DuPont (Wilmington, DE) and Parylene C dimer and Silane A174 from Specialty Coating Systems (Indianapolis, IN).

### Macroscale cell culture

All cell lines used in this study were purchased from ATCC (Manassas, VA). NIH-3T3 cells were grown in DMEM containing 100 U ml^−1^ penicillin G and 100 μg ml^−1^ streptomycin supplemented with 10% calf serum (CS) in a humidified incubator at 37 °C with 5% CO_2_. The MCF-7 and MDA-MB-231 cell lines were grown in DMEM containing 100 U ml^−1^ penicillin G and 100 μg ml^−1^ streptomycin supplemented with 10% fetal bovine serum (FBS) in a humidified incubator at 37 °C with 5% CO_2_. With the exception of the cell density optimization studies, all DMF experiments were seeded with a cell density of 500,000 cells per ml.

### On-chip reagent composition

On-chip cell culture reagents including culture media (10% serum (CS or FBS) and 0.05% v/v Pluronic F68), starving media (0.5% serum (CS or FBS) and 0.05% v/v F68) and serum-free media (0.05% v/v F68) were formed in DMEM containing 100 U ml^−1^ penicillin G and 100 μg ml^−1^ streptomycin. Immunocytochemistry reagents including fixing solution (FS) (4% paraformaldehyde and 0.05% v/v F68), WB1 (0.05% v/v F68), WB2 (0.05% v/v Brij 35), blocking solution (BS) (1% bovine non-fat dried milk and 0.05% v/v Brij 35) and permeabilization solution (PS) (0.2% v/v Tween-20) were formed in DPBS. Primary antibody staining solutions for Akt (comprising pan Akt (Mouse mAb, 1:50, CST 2920S) and p-Akt S473 (Rabbit mAb, 1:200, CST 4060P)), PDGR-β (comprising PDGR-β (Mouse mAb, 1:50, CST sc-19995) and p-PDGR-β Y857 (Rabbit pAb, 1:200, SCB sc-12907-R) or p-PDGR-β Y1021 (Rabbit pAb, 1:200, SCB sc-12909-R)), and GAPDH (Rabbit pAb, 2 μg ml^−1^, SCB sc-25778) were formed in BS. Secondary antibody staining solution, comprising Anti-Mouse IgG Alexa Fluor 647 (Fab2, 1:500, CST 4410S) and Anti-Rabbit IgG Alexa Fluor 555 (Fab2, 1:500, CST 4413S) was formed in BS. Secondary antibody staining solution for signal/background optimization, containing the Anti-Rabbit IgG Cy3 (Goat pAB, 1:100, Sigma-Aldrich C2306) was formed in BS.

### Device fabrication and operation

DMF devices, each comprising a bottom plate and top plate, were fabricated in the University of Toronto Nanofabrication Centre (TNFC) clean room facility, using transparent photomasks printed at 20,000 d.p.i. (Pacific Arts and Designs Inc., Markham, ON). The bottom plates of DMF devices bearing an array of electrodes were formed by photolithography and wet etching. Briefly, chromium (200-nm thick) and photoresist-coated glass substrates (2′′ × 3′′ × 1.1 mm) (Telic Co., Santa Clarita, CA) were exposed to UV light through a photomask using a Suss MicroTec mask aligner (29.8 mW cm^−2^, 10 s). The exposed substrates were developed in MF-321 (3 min), etched in CR-4 (3 min) and the remaining photoresist was stripped in AZ-300T (5 min). After forming electrodes, the substrates were primed for parylene coating by immersing in silane solution (2-propanol, DI water and A174 50:50:1 v/v/v, 10 min) and curing on a hot plate (80 ^o^C, 10 min). After rinsing and drying, contact pads of the devices were covered with dicing tape (Semiconductor Equipment Corp., Moorpark, CA). Next, the devices were coated with ∼7 μm of Parylene C (vapour deposition) and ∼200 nm of Teflon-AF (spin-coating, 1% w/w in Fluorinert FC-40, 2000, r.p.m., 60 s), and post baked on a hot plate (165 °C, 10 min). The polymer coatings were removed from contact pads by removing the dicing tape.

The top plates of DMF devices, bearing hydrophilic sites for cell culture, were formed by a modification of a previously reported Teflon-AF lift-off technique^34^ on indium-tin oxide (ITO) coated glass substrates (Delta Technologies Ltd, Stillwater, MN). Prior to fabrication, ITO substrates (3′′ × 1′′) were immersed in a cleaning solution comprising DI water, NH_4_OH, and H_2_O_2_ (5:1:1 v/v/v) on a hot plate (30 min, 80 ^o^C). After thorough rinsing with DI water and drying with nitrogen gas, S1811 was spin-coated (3,000 r.p.m., 45 s) on ITO substrates and baked on a hot plate (2 min, 95 °C). The photoresist-coated ITO substrates were exposed to UV light through a photomask using a Suss MicroTec mask aligner (29.8 mW cm^−2^, 10 s). The exposed substrates were developed in MF-321 (3 min), rinsed with DI water and dried under a gentle stream of nitrogen. Subsequently, the developed substrates were exposed (without photomask) to UV light (29.8 mW cm^−2^, 10 s). After forming the photoresist patterns, the substrates were spin-coated with Teflon-AF (1% wt/wt in FC-40, 4,000 r.p.m., 30 s), baked on a hot plate (2 min, 165 °C) and immersed in acetone until lift-off occurred (∼5–10 s), exposing a pattern of ITO spots.

Prior to assembly of a DMF device, one pair of top and bottom plates was sterilized by immersing in 70% ethanol (10 min) and then air dried. The hydrophilic sites (exposed ITO) on the top plate were aligned visually to the center of the electrode array on bottom plate, and the two plates were joined by a spacer formed from two pieces of Scotch double-sided tape (3M, St. Paul, MN) or double coated white polyester diagnostic tape 9965 (3M) with total spacer thickness of ∼180 μm. The bottom plate device design featured an array of 80 actuation electrodes (2.2 × 2.2 mm ea.) connected reservoir electrodes for reagent storage and waste removal. The actuation electrodes were roughly square with interdigitated borders (140 μm peak-to-peak sinusoids) and inter-electrode gaps of 30–80 μm. Unit droplet and reservoir droplet volumes on these devices were ∼900 nl and ∼6 μl, respectively. The top plate design featured a row of eight circular hydrophilic spots (1.75 mm diameter, 4.4 mm pitch). The VMs dispensed on these lift-off spots were ∼400 nl.

An in-house designed computer-controlled instrument^55^ powered by DropBot^51^, an open-source DMF automation system (with software, schematics, CAD files, parts lists, assembly instructions and more available at http://microfluidics.utoronto.ca/dropbot), was used to manage and record droplet operations. Droplets were actuated by applying a preprogrammed sequence of driving voltages (80–100 *V*_RMS_ 10 kHz sine wave) between the top plate (ground) and electrodes in the bottom plate through a Pogo pin interface (90 pins). Reagents were loaded to their respective reservoirs by pipetting the reagent adjacent to the gap between the bottom and top plates and actuating the reservoir electrodes. Once inside the reservoirs, droplets were actively dispensed by electrostatically stretching the droplets from the reservoirs followed by splitting. In addition to active dispensing, the devices also relied on a phenomenon known as passive dispensing. To initiate passive dispensing, a source droplet is translated across a vacant lift-off spot and a sub-droplet or ‘VM' is spontaneously formed because of hydrophobic–hydrophilic interactions. Once a VM is formed, subsequent passive dispensing will displace the contents of the VM with the contents of the new source droplet. Waste and unused fluids were removed by delivering them to reservoirs containing KimWipes (Kimberly-Clark, Irving, TX).

### DMF cell culture and immunocytochemistry

DMF was used to automate the protocols required for cell culture and immunocytochemistry including cell seeding, starving, ligand stimulation, fixation, permeabilization, blocking, staining and washing. In all droplet manipulation steps, the device was oriented in standard configuration, with the top plate on top; in all incubation steps, the device was inverted, with the top plate on bottom. To seed cells, 2.7 μl (3 × 900 nl) of cells suspended in culture media were sequentially passively dispensed on each vacant lift-off spot, forming VMs of ∼400 nl. The device was inverted and incubated in a homemade humidified chamber at 37 ^o^C for 24 h, allowing cells to settle and adhere to the hydrophilic sites. In the next 11 steps, a sequence of reagents and WBs was sequentially passively dispensed on each VM; unless otherwise stated, droplet operations and incubations were performed in room temperature. (1) Cells were starved by passively dispensing 5.4 μl (6 × 900 nl) of starving media and incubating at 37 ^o^C for 24 h. (2) The starving media was rinsed by passively dispensing 5.4 μl (6 × 900 nl) of serum-free DMEM. (3) Cells were stimulated by passively dispensing 1.8 μl (2 × 900 nl) containing PDGF-BB in serum-free DMEM; the volume, concentration, frequency and duration were varied depending on the experiment. (4) Immediately after stimulation, cells were fixed by passively dispensing 2.7 μl (3 × 900 nl) of FS and incubating for 10 min. (5) The FS was rinsed by passively dispensing 5.4 μl (6 × 900 nl) of WB1. (6) The fixed cells were permeabilized by passively dispensing 2.7 μl (3 × 900 nl) of PS and incubating for 10 min. (7) The PS was rinsed by passively dispensing 8.1 μl (9 × 900 nl) of WB2. (8) The rinsed cells were blocked by passively dispensing 2.7 μl (3 × 900 nl) of BS and incubating for 1 h. (9) The blocked cells were stained with primary antibodies by passively dispensing 2.7 μl (3 × 900 nl) of primary antibody solution and incubating overnight in 4 ^o^C. (10) The unbound primary antibodies were removed by passively dispensing 5.4 μl (6 × 900 nl) of WB2. (11) The primary antibodies were stained by secondary antibodies by passively dispensing 2.7 μl (3 × 900 nl) of secondary antibody solution and incubating for 2 h. Subsequently, the top plate was dissembled from the device, rinsed in a falcon tube containing 10 × dilution of 0.5% Tween 20 in DPBS for 30 s, rinsed in another falcon tube containing DI water for 30 s and air dried in the dark. In time-series experiments, droplet sequences in step 3 were staggered so that step 4 was performed simultaneously for all VMs in one device—this prevents cells from being exposed to paraformaldehyde fumes in step 3.

### Microarray scanner analysis and Cellprofiler pipeline

Top plates bearing stained cells were analysed in an Axon GenePix 4000B microarray scanner (Sunnyvale, CA). Typically, the scanner was set at 5 μm resolution, 0 μm focus, with 532 nm and 635 nm excitation lasers set at 100% power and the photomultiplier (PMT) potentials set at 500 V. These settings were adjusted accordingly to maximize signal without saturating the PMTs. For example, in some instances, the power of the 532 nm laser was decreased to 10% to prevent signal saturation. In other instances, the PMT voltage for the Cy5 channel was increased to 600 V when the signal was weak.

Microarray scans were cropped in ImageJ and analysed using the open-source CellProfiler 2.0 r11710 software package (http://www.cellprofiler.org/). A custom pipeline was developed, including image cropping, identifying single-cell regions-of-interest from whole Akt or PDGFR fluorescent images (Cy5 channel), measuring size and shape of the cells, analysing cell neighbours, and measuring fluorescence intensities of both phosphorylated (Cy3 channel) and non-phosphorylated (Cy5 channel) Akt or PDGFR. To identify cells, the software was instructed to detect 15–100 μm diameter objects using the Otsu Global thresholding method (two classes, weighted variance, 0.7 threshold correction factor). For each cell, the fluorescence intensity from the phosphorylated protein was normalized to the fluorescence intensity from the corresponding whole protein (phosphorylated+non-phosphorylated) to account for differences in single-cell protein expression level. To express as fold change, the each data point was further normalized to the average signal from negative control cells on the same device, which were exposed to a droplet containing only serum-free DMEM (that is, no stimulation). These measures allowed for accumulation of comparable data collected on different days (Supplementary Fig. 18). Bulk response plots, histograms and scatter plots were generated using GraphPad Prism software (GraphPad, La Jolla, CA).

In some experiments, the software was used to calculate the eccentricity and form factor of each cell. Eccentricity is the ratio of the distance between the foci of a fitted ellipse and its major axis length. The value is between 0 and 1, with 0 eccentricity representing a perfectly circular object and 1 eccentricity representing a line segment. Form factor is calculated as 4*π* × Area/Perimeter^2^, with a value of 1 representing a perfectly circular object. The software was also used to determine the position of each cell, the distance of the closest neighbour for each cell and the number of neighbours within 125 μm distance from each cell. The distance of 125 μm represents the estimated diffusion distance of a PDGF-BB molecule in 5 min.

### Washing efficiency

The washing efficiency of passive dispensing was evaluated using a soluble fluorescent tracker. First, an on-chip calibration curve was generated using various concentrations of fluoresceinamine (5, 0.5, 0.05, 0.005 and 0 μM) in WB1. These solutions were passively dispensed on vacant lift-off spots to form ∼400 nl VMs. The fluorescent intensities in the VMs were measured using a Typhoon 9400 (GE Healthcare Bio-Sciences, Pittsburgh, PA) scanner using 520 BP 40 emission filter, blue laser, +3 mm focus and 450 V PMT voltage. Second (in separate experiments), 5 μM fluoresceinamine was passively dispensed onto vacant lift-off spots and measured as above (wash 0). Next, 1.8 μl (2 × 900 nl) of WB1 were passively dispensed across each lift-off site and the resulting (diluted) VMs were measured as above (wash 1). This was repeated three times (wash 2, 3 and 4), and the fluorescence intensities were extrapolated to fluoresceinamine concentration using the calibration curve. Finally, the concentrations were normalized to the maximum concentration and plotted against the wash number.

### Optimization experiments

The method was optimized for cell density and signal/background ratio using a variation of the 11-step protocol on NIH-3T3 cells. In cell density optimization experiments, prior to step 1, cell suspensions at different densities (10^3^, 10^4^, 10^5^ and 10^6^ cells per ml) were seeded and cultured overnight as described above. The cells were imaged in brightfield using a × 20 objective on a Leica DM2000 microscope (Leica Microsystems, Inc., Concord, ON, Canada). Steps 1–3 were omitted, and in steps 9 and 11 cells were stained with GADPH primary antibody and anti-Rabbit IgG Cy3 secondary antibody. For signal/background optimization, prior to step 1, cell suspensions at 10^6^ cells per ml were seeded and incubated overnight as above. Steps 1–3 were omitted, and in steps 9 and 11 cells were stained with GAPDH primary antibodies and anti-Rabbit IgG Cy3 secondary antibodies. Control experiments were identical, except that BS was used in place of primary antibodies in step 9. In all experiments after optimization, cells were seeded at a density of 500,000 cells per ml, yielding ∼150–300 cells in each VM.

### Dose–response experiments

PDGF-BB dose–response curves were collected for PDGFR (both Y857 and Y1021) and Akt using the 11-step protocol on NIH-3T3 cells. In step 3, 1.8μl (2 × 900 nl) of PDGF-BB (0, 3.125, 6.25, 12.5, 25, 50 and 200 ng ml^−1^) were passively dispensed to VMs and incubated for 2 min (for PDGFR) or 5 min (Akt) prior to continuing with step 4. In step 9, cells were labelled with primary antibodies for PDGR-β (Mouse) and p-PDGR-β (Rabbit) or for Akt (Mouse) and p-Akt (Rabbit). In step 11, cells were stained with secondary antibodies including anti-Mouse IgG Alexa Fluor 647 and anti-Rabbit IgG Alexa Fluor 555. To determine the Hill coefficient, the average signal from negative control cells was subtracted from the average signal from cells stimulated by various concentration of PDGF-BB on the same device. The background subtracted data was normalized to the highest average response on the same device. The normalized, background subtracted data was fit to a Hill function:


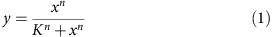


where *y* is the proportion of activated phosphorylation site, *x* is the concentration of PDGF-BB, *K* is the concentration of PDGF-BB required for half maximal phosphorylation, and *n* is the Hill coefficient, describing the cooperativity of ligand-induced activation of receptor.

### Droplet movement effects on cell signalling

The effects of droplet movement were tested by probing Akt phosphorylation using the 11-step protocol on NIH-3T3 cells. In step 3, 1.8 μl (2 × 900 nl) of 0 ng ml^−1^ PDGF-BB were delivered to VMs and moved continuously, back-and-forth over the cells for 75 times over 5 min. For comparison, 1.8 μl (2 × 900 nl) of 0 ng ml^−1^ (negative control) or 3.125 ng ml^−1^ (positive control) were passively dispensed to VMs and incubated with no droplet movement for 5 min prior to continuing with step 4. In step 9, cells were labelled with primary antibodies for Akt (Mouse) and p-Akt (Rabbit); in step 11, cells were stained with secondary antibodies including anti-Mouse IgG Alexa Fluor 647 and anti-Rabbit IgG Alexa Fluor 555. Using GraphPad Prism software, a two-tailed Student's *t*-test (95% confidence intervals) was used to compare the continuous mixing and negative control experiments.

### Breast cancer cell time series

Time-series responses were collected for Akt using the 11-step protocol on MCF-7 and MDA-MB-231 breast cancer cell lines. In step 3, 1.8 μl (2 × 900 nl) of 50 ng ml^−1^ PDGF-BB were passively dispensed to VMs and incubated for various duration (0, 1, 2, 3, 4, 5, 6 or 7 min) prior to continuing with step 4. In step 9, cells were labelled with primary antibodies for Akt (Mouse) and p-Akt (Rabbit); in step 11, cells were stained with secondary antibodies including anti-Mouse IgG Alexa Fluor 647 and anti-Rabbit IgG Alexa Fluor 555. In the average response plot, a two-way analysis of variance with Bonferroni post test was applied to compare means between multiple time points. The hyperactive responders were defined as the population of cells that have a p-Akt (fold change) greater than that of the 75.0% quartile plus 1.5 times the inter quartile range (the 75.0% quartile minus 25.0% quartile), which were determined by plotting the p-Akt (fold change) in an outlier plot using JMP 10 (SAS Institute Inc., Cary, NC). Contour plots of Akt phosphorylation as a function of position were plotted using JMP 10. 2D profile heat maps were created in MATLAB v2009a (MathWorks) using the dscatter function (creator: R. Henson, MathWorks File Exchange). A two-tailed Mann–Whitney test (95% confidence intervals) was used to compare the parameters measured by Cellprofiler. Data were considered statistically significant at *P*<0.05. Statistical analysis was performed with GraphPad Prism software.

### High- and low-concentration ligand single-bolus time series

High- and low-stimulation concentration/time-series responses were collected for PDGFR Y857 and Y1021 using the 11-step protocol on NIH-3T3 cells. In step 3, 1.8μl (2 × 900 nl) of 3.125 or 50 ng ml^−1^ PDGF-BB were passively dispensed to the VMs and incubated for various durations (0, 30, 45, 60, 75, 90, 105 or 120 s) prior to continuing with step 4. In step 9, cells were labelled with primary antibodies for PDGR-β (Mouse) and p-PDGR-β (Rabbit); in step 11, cells were stained with secondary antibodies including anti-Mouse IgG Alexa Fluor 647 and anti-Rabbit IgG Alexa Fluor 555.

### Inhibitor single-bolus time series

Inhibitor time-series responses were collected for PDGFR Y857 using a variation of the 11-step protocol on NIH-3T3 cells. In step 2, the starved cells were rinsed in serum-free DMEM supplemented with 10 mM sodium orthovanadate, and in step 3 cells were stimulated with 1.8μl (2 × 900 nl) of 3.125 ng ml^−1^ PDGF-BB supplemented with 10 mM sodium orthovanadate and were allowed to incubate for various durations (0, 120, 180, 240, 300, 360 or 420 s) prior to continuing with step 4. In control experiments, sodium orthovanadate was not included in the rinsing and stimulation reagents. In step 9, cells were labelled with primary antibodies for PDGR-β (Mouse) and p-PDGR-β (Rabbit); in step 11, cells were stained with secondary antibodies including anti-Mouse IgG Alexa Fluor 647 and anti-Rabbit IgG Alexa Fluor 555. A two-way analysis of variance with Bonferroni post test was applied to compare means between multiple time points.

### Ligand double-bolus time series

Ligand renewal time-series responses were collected for PDGFR Y857 using a variation of the 11-step protocol on NIH-3T3 cells. The standard step 3 was replaced with step 3a and step 3b. In step 3a, cells were stimulated with 1.8 μl (2 × 900 nl) of 3.125 ng ml^−1^ or 0 ng ml^−1^ (vehicle) PDGF-BB and were allowed to incubate for 90 s. In step 3b, cells were stimulated again with 1.8 μl (2 × 900 nl) of 3.125 ng ml^−1^ PDGF-BB and were allowed to incubate for an additional duration (30, 90, 150, 270 or 330 s) prior to continuing with step 4. In reference single-bolus experiments, only step 3a was performed with 1.8 μl (2 × 900 nl) of 3.125 or 6.25 ng ml^−1^ PDGF-BB, followed by step 4. In step 9, cells were labelled with primary antibodies for PDGR-β (Mouse) and p-PDGR-β (Rabbit); in step 11, cells were stained with secondary antibodies including anti-Mouse IgG Alexa Fluor 647 and anti-Rabbit IgG Alexa Fluor 555.

### Ligand pulse experiments

Ligand pulse responses (that is, signals measured after stimulation and wash) were collected for PDGFR Y857 and Akt S473 using a variation of the 11-step protocol on NIH-3T3 cells. The standard step 3 was replaced with step 3i and step 3ii. In step 3i, cells were stimulated with 1.8 μl (2 × 900 nl) of 3.125 ng ml^−1^ PDGF-BB and were allowed to incubate for 10 or 30 s for PDGFR Y857, or for 3, 10, 20, 30 or 40 s for Akt S473. In step 3ii, the cells were washed by delivering 1.8 μl (2 × 900 nl) of ligand-free medium and allowed to incubate for an additional duration (for a total duration of 2 min for PDGFR experiments, or total durations of 1, 2, 3, 4 and 5 min for Akt experiments) prior to continuing with step 4. In control experiments, the standard step 3 was performed, either with serum-free medium (negative control) or 3.125 ng ml^−1^ PDGF-BB (positive control), followed by incubation for 2 min (for PDGFR) or 5 min (for Akt) prior to continuing with step 4. (The timing of each pulse experiment is represented in the experimental schemes on top of each panel in Fig. 6.) In step 9, cells were labelled with primary antibodies for PDGR-β (Mouse) and p-PDGR-β (Rabbit) or for Akt (Mouse) and p-Akt (Rabbit); in step 11, cells were stained with secondary antibodies including anti-Mouse IgG Alexa Fluor 647 and anti-Rabbit IgG Alexa Fluor 555.

### Live-cell imaging

MDA-MB-231 cells (500,000 cells per ml) were seeded, incubated overnight and starved overnight using DMF. The cells were stained by passively dispensing 2.7 μl (3 × 900 nl) of cell tracer (CFDA SE dye, 1 μM in WB, Life Technologies) and incubating for 15 min at 37 ^o^C. Excess dye was quenched by passively dispensing 2.7 μl (3 × 900 nl) of starving media and incubating for at least 30 min. The stained cells were stimulated by merging a 1.8 μl (2 × 900 nl) of PDGF-BB (200 ng ml^−1^ in serum-free media) to the VM and imaged by confocal microscopy (Nikon A1, Nikon Instruments Inc, Melville, NY) using Plan fluor ELWD × 40 (numerical aperture 0.60, WD 3.6 - 2.8 mm) objective and fluorescein filter cube. One minute after stimulation, time lapsed images of live cells were captured for 30 min, at 1 frame every 30 s.

## Additional information

**How to cite this article:** Ng, A. H. C *et al.* Digital microfluidic immunocytochemistry in single cells. *Nat. Commun.* 6:7513 doi: 10.1038/ncomms8513 (2015).

## Supplementary Material

Supplementary Information Supplementary Figures 1-18, Supplementary Note 1.

Supplementary Movie 1 Movie depicting formation of eight virtual microwells on hydrophilic spots by passive dispensing.

Supplementary Movie 2 Movie illustrating reagent delivery (blue dye) and solution exchange on four virtual microwells by passive dispensing.

Supplementary Movie 3 Movie depicting passive dispensing of three successive 1.8-microliter droplets to a virtual microwell. Approximately 1 second is required between two reagent exchanges.

Supplementary Movie 4 Fluorescent time-lapse video of live MDA-MB-231 cells stained with cell tracer after stimulation by PDGF-BB.
