# Supplementary material for: Digital microfluidic immunocytochemistry in single cells
Source: Nat Commun. 2015 Jun 24;6:7513. doi: 10.1038/ncomms8513 (PMC4491823; doi:10.1038/ncomms8513)
Supplement: Supplementary Information — Supplementary Figures 1-18, Supplementary Note 1. [file ncomms8513-s1.pdf]

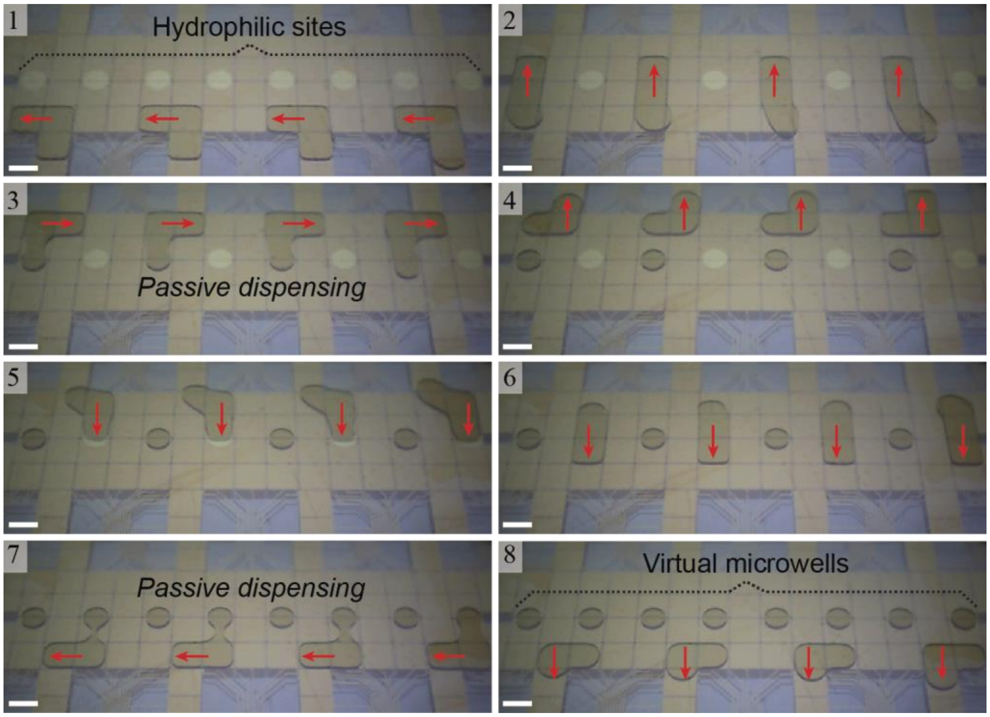

Supplementary Figure 1: Formation of virtual microwells. Video sequence from **Supplementary Movie 1** (frames 1-8) illustrating the formation of eight virtual microwells by passive dispensing, a surface tension-driven phenomenon in which a sub-droplet is dispensed on a hydrophilic site when a source droplet is translated across the site. Red arrows indicate the direction each droplet is moving. Scale bars, 2 mm.

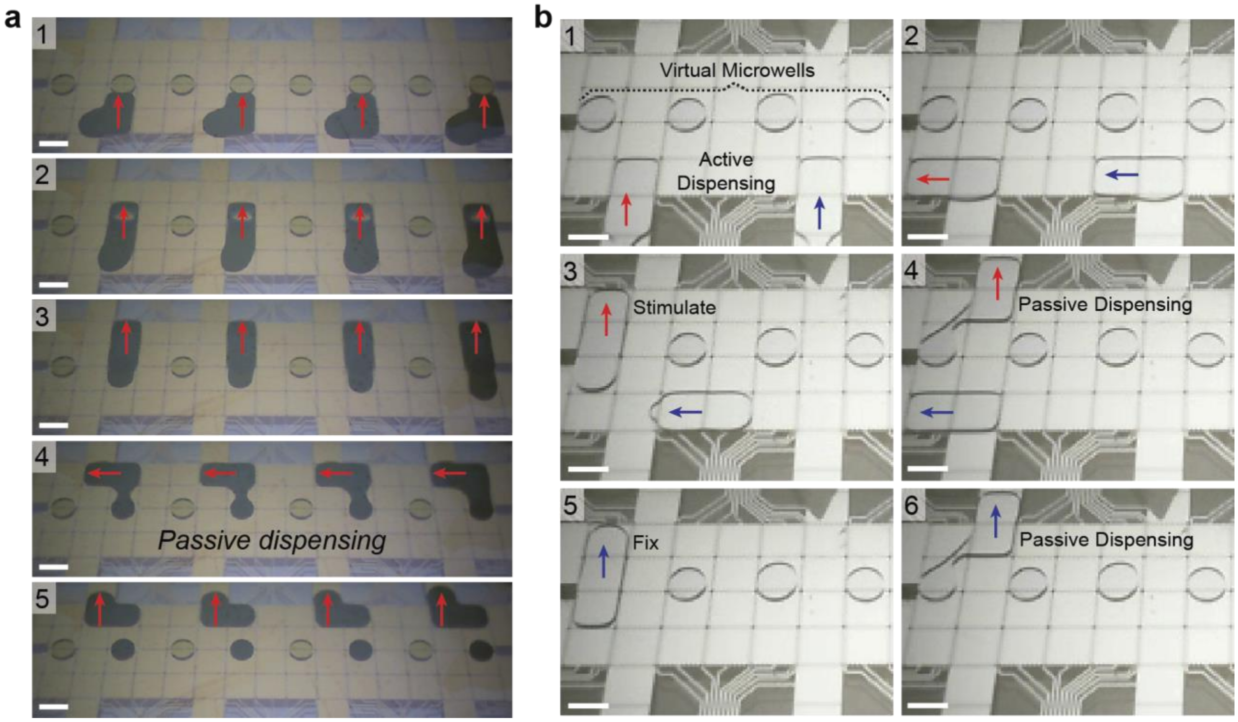

Supplementary Figure 2: Reagent exchange by passive dispensing. (**a**) Video sequence (frames 1-5) from **Supplementary Movie 2** illustrating reagent delivery and solution exchange on four virtual microwells (VMs) by passive dispensing. The initial solution in the are is transparent; blue dye was added to the displacing solution for visualization. Red arrows indicate the direction each droplet is moving. Scale bars, 2 mm. (**b**) Video sequence (frames 1-6) depicting passive dispensing of two successive 1.8 μL droplets to a virtual microwell. Red and blue arrows indicate the directions that the first reagent and second reagent droplets are moving, respectively. Scale bars, 2 mm.

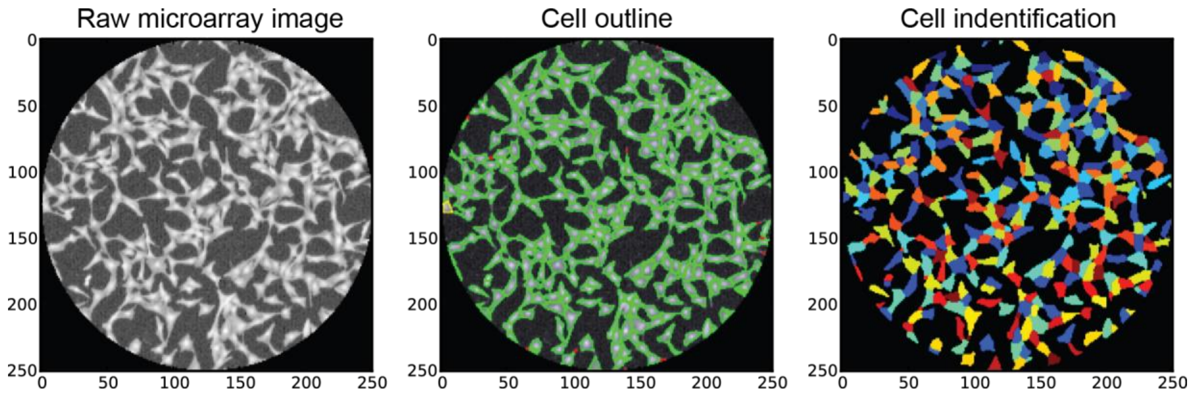

Supplementary Figure 3: Automated image analysis procedure. The gray-scale picture (left) is a representative cropped region of a virtual microwell (250 pixel diameter) from a raw microarray scan. Each pixel is 5 × 5µm, corresponding to the scanner resolution. A custom routine was written in CellProfiler (http://www.cellprofiler.org/) to find the cell borders (middle), and to identify the single-cell regions of interest (right).

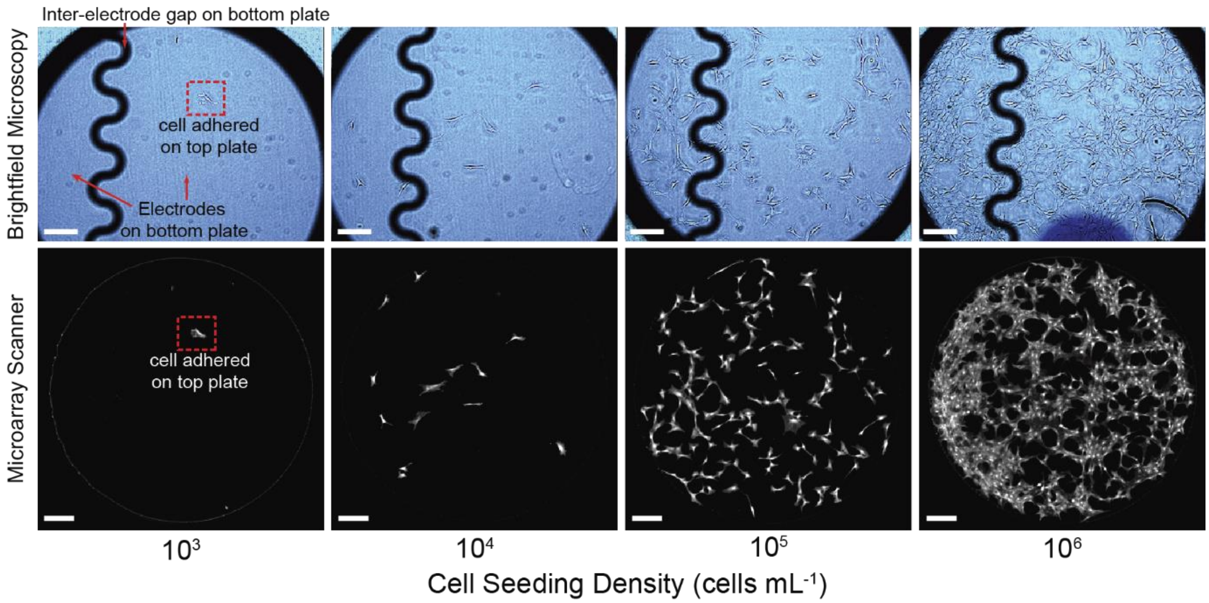

Supplementary Figure 4: Brightfield microscopy (top) and microarray scans (bottom) of NIH 3T3 fibroblasts seeded at various densities on DISC devices. In the top images, it is apparent that cells are adhered to the transparent top plate; DMF driving electrodes (with sinusoidal gaps between them) can be seen on the bottom plate. Scale bars, 100 µm (top) and 200 µm (bottom).

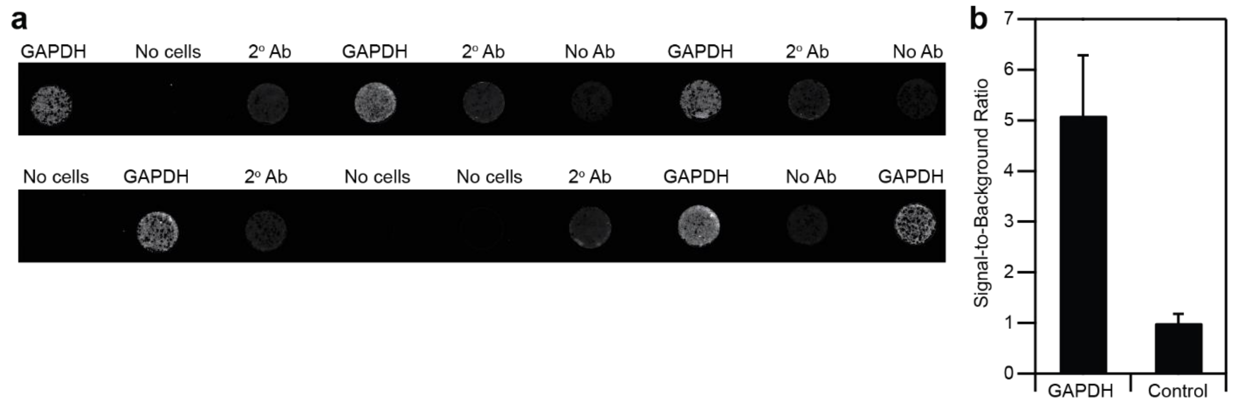

Supplementary Figure 5: Signal-to-background characterization of stained cells. (**a**) Microarray scans of virtual microwells bearing cells stained for GAPDH (1° antibody for GAPDH and 2° antibody), cells stained with 2° antibody only (2° Ab), cells that were not stained (no Ab), and no cells. (**b**) Bar graph of GAPDH staining and control (2° antibody only) showing the signal-to-background ratio (averaged across each virtual microwell) of the optimized DISC protocol. Error bars are +/- 1 S.D. from at least 5 technical replicates.

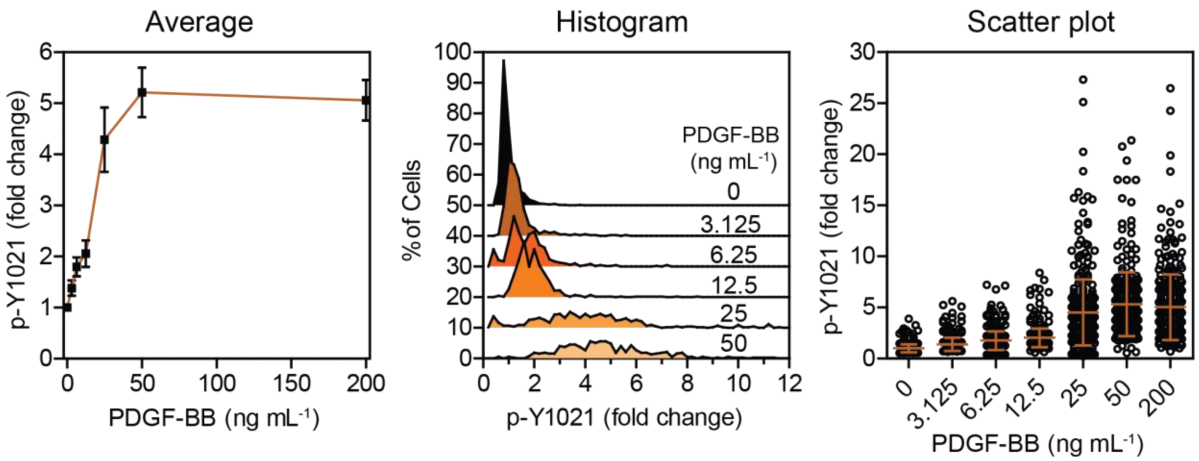

Supplementary Figure 6: Dose-response plots of PDGFR Y1021 phosphorylation 2 min after stimulation. Data are expressed as fold changes relative to the response of cells exposed to a droplet containing only serum-free DMEM (i.e., no stimulation). The 'Average' plots (left) are average response +/- S.E.M. from an average of all of the cells in 3-4 virtual microwells performed on different days. Representative 'Histograms' (middle) and single cell 'Scatter plots' (right) with population mean +/- 1 S.D. (representing a total of 371-627 cells per condition) reveal the effect of PDGF-BB dosage on cell population distribution. Histogram distributions were offset vertically for comparison.

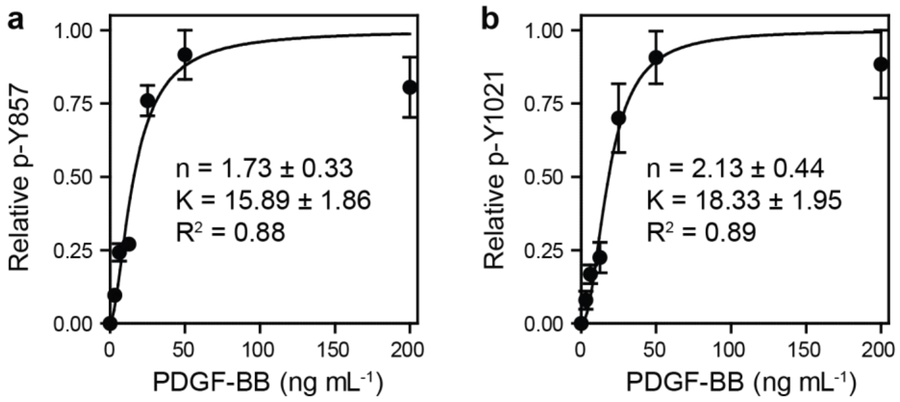

Supplementary Figure 7: PDGF receptor phosphorylation cooperativity. Data from Figure 3b/left panel (main text) and **Supplementary Figure 6, left panel** was fit to equation 1 in the main text (solid curve) for PDGFR Y857 (**a**) and PDGFR Y1021 (**b**) with indicated Hill coefficient (n), half-maximum phosphorylation concencentration in ng mL^{-1} (K), and goodness of fit of non-linear regression (R^2). Error bars are +/- 1 S.E.M. from 3 different day replicates.

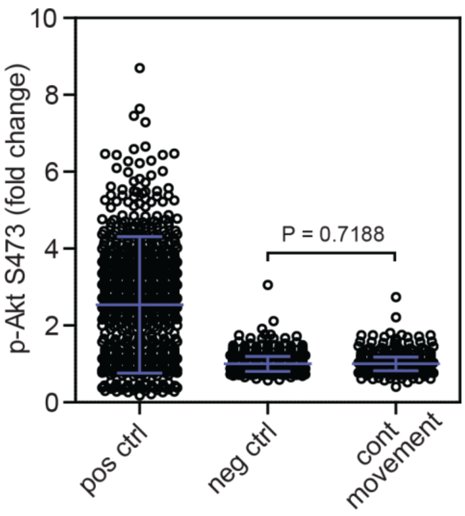

Supplementary Figure 8: Effects of droplet movement on cell signaling. Scatter plot of Akt S473 phosphorylation in NIH 3T3 cells 5 min after stimulation by 3.125 ng mL^{-1} PDGF-BB (pos ctrl), 0 ng mL^{-1} PDGF-BB (neg ctrl), and 0 ng mL^{-1} PDGF-BB with continuous back-and-forth droplet movement (comprising ~75 passes) over the cells for 5 minutes (cont. movement). Data are expressed as fold changes relative to neg ctrl. Error bars represent population mean +/- 1 S.D., representing a total of 938-1055 cells per condition from 8-12 virtual microwells performed on different days.

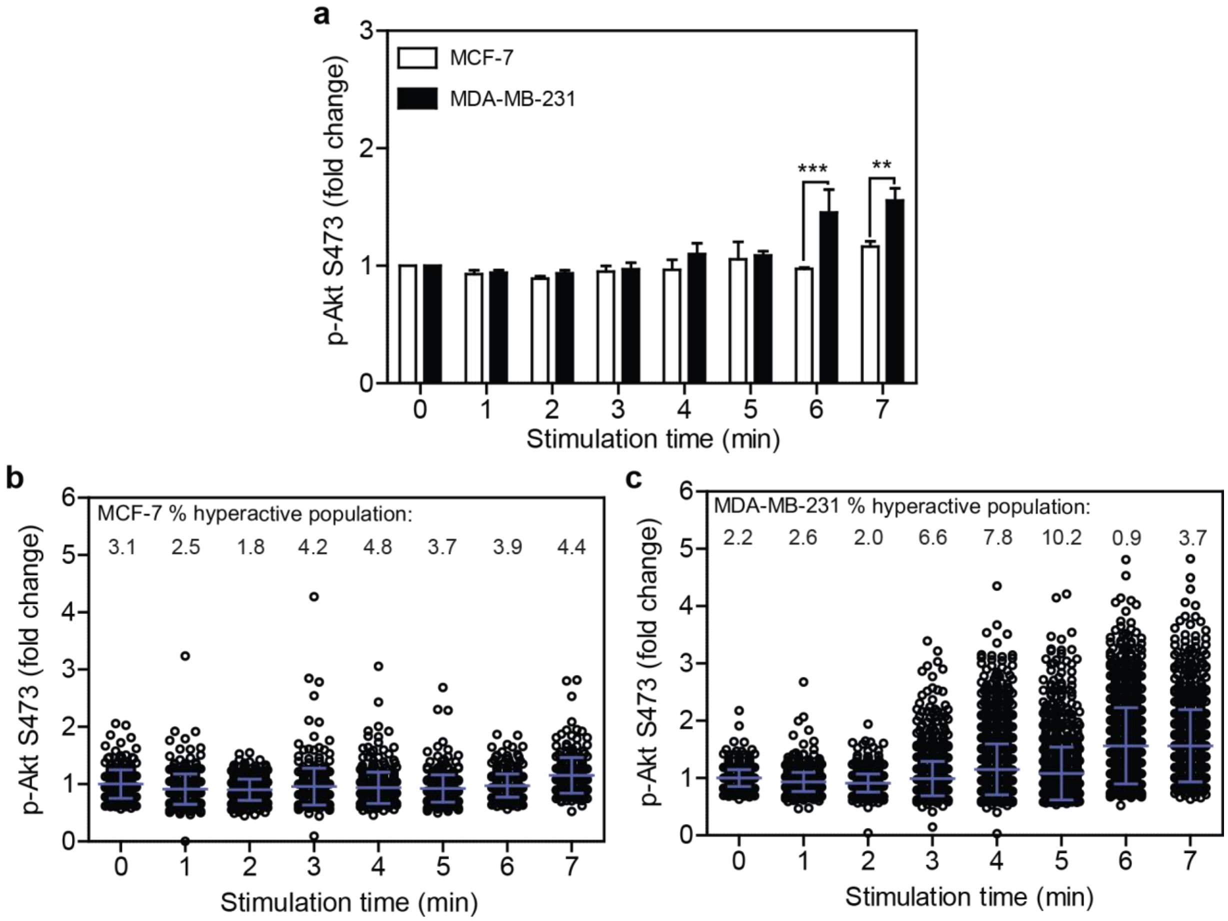

Supplementary Figure 9: Time-resolved DISC results for Akt phosphorylation in breast cancer cell lines stimulated by PDGF-BB. (**a**) Plot showing average Akt S473 phosphorylation status in MCF-7 and MDA-MB231 cell lines stimulated for 0-7 min with 50 ng mL^{-1} of PDGF-BB. Error bars are +/- S.E.M. from an average of all of the cells in 3 virtual microwells performed on different days; **$p<0.01$, ***$p<0.001$. Data were considered statistically significant at $p<0.05$. Corresponding single-cell scatter plots for MCF-7 (**b**) and MDA-MB-231 (**c**) cell lines. The percentage of hyperactive cells are indicated above each group of scatter data. Error bars are population mean +/- S.D. and scatter plots were generated from at least 3 different day replicates (representing a total of 271-2219 cells per condition).

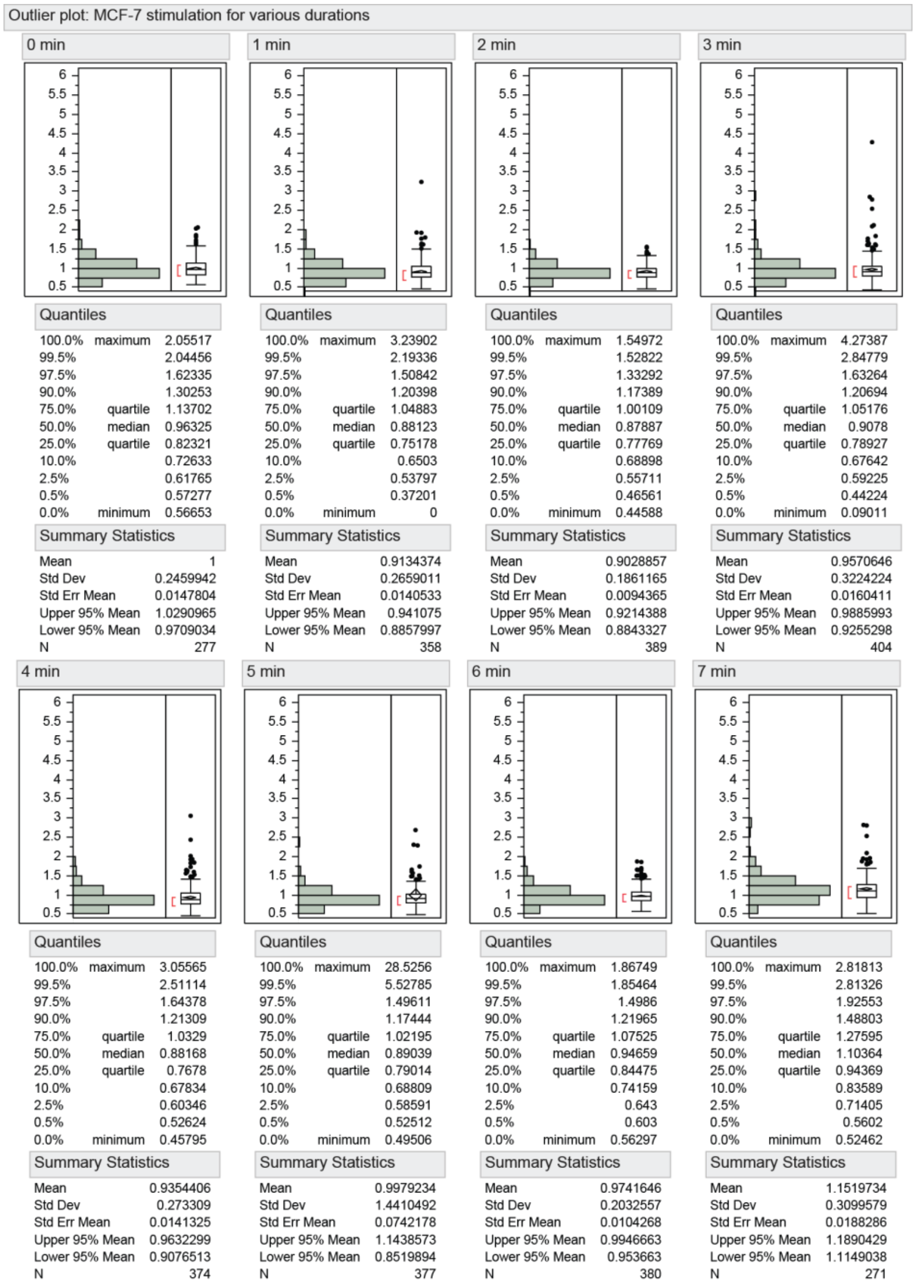

Supplementary Figure 10: Outlier box plots of time-resolved Akt phosphorylation in MCF-7 cells after stimulation with 50 ng mL^{-1} of PDGF-BB for 0-7 min. The box represents the inter-quartile range (IQR, 75.0% quartile minus the 25.0% quartile) and the errors bars (whiskers) represent + or - 1.5×IQR from the 3rd or 1st quartile, respectively. Cells with responses greater than 75.0% quartile plus 1.5×IQR were considered hyperactive responders.

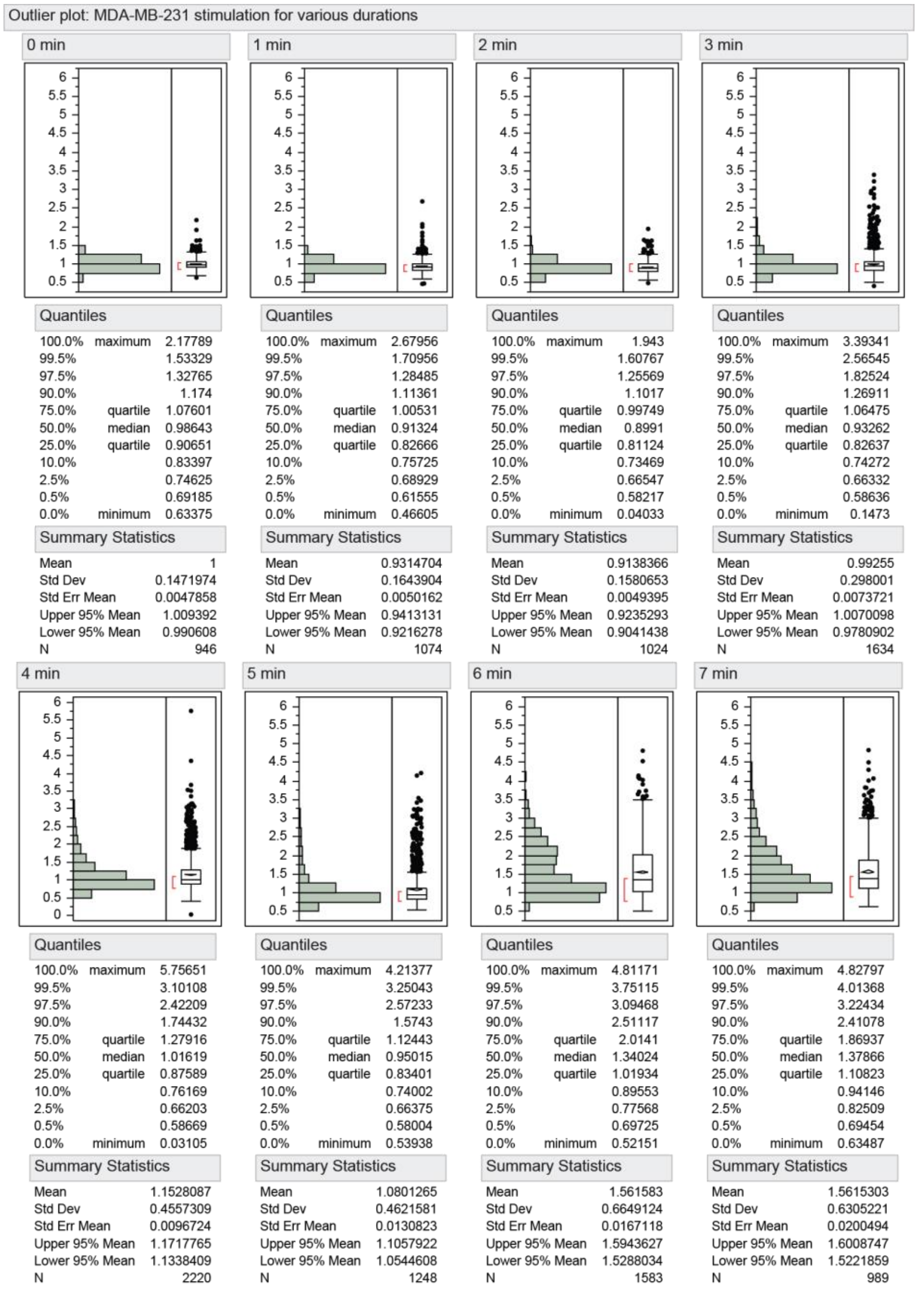

Quantiles

		0 min	1 min	2 min	3 min	4 min	5 min	6 min	7 min
100.0%	maximum	2.17789	2.67956	1.943	3.39341	5.75651	4.21377	4.81171	4.82797
99.5%		1.53329	1.70956	1.60767	2.56545	3.10108	3.25043	3.75115	4.01368
97.5%		1.32765	1.28485	1.25569	1.82524	2.42209	2.57233	3.09468	3.22434
90.0%		1.174	1.11361	1.1017	1.26911	1.74432	1.5743	2.51117	2.41078
75.0%	quartile	1.07601	1.00531	0.99749	1.06475	1.27916	1.12443	2.0141	1.86937
50.0%	median	0.98643	0.91324	0.8991	0.93262	1.01619	0.95015	1.34024	1.37866
25.0%	quartile	0.90651	0.82666	0.81124	0.82637	0.87589	0.83401	1.01934	1.10823
10.0%		0.83397	0.75725	0.73469	0.74272	0.76169	0.74002	0.89553	0.94146
2.5%		0.74625	0.68929	0.66547	0.66332	0.66203	0.66375	0.77568	0.82509
0.5%		0.69185	0.61555	0.58217	0.58636	0.58669	0.58004	0.69725	0.69454
0.0%	minimum	0.63375	0.46605	0.04033	0.1473	0.03105	0.53938	0.52151	0.63487

Summary Statistics

	0 min	1 min	2 min	3 min	4 min	5 min	6 min	7 min
Mean	1	0.9314704	0.9138366	0.99255	1.1528087	1.0801265	1.561583	1.5615303
Std Dev	0.1471974	0.1643904	0.1580653	0.298001	0.4557309	0.4621581	0.6649124	0.6305221
Std Err Mean	0.0047858	0.0050162	0.0049395	0.0073721	0.0096724	0.0130823	0.0167118	0.0200494
Upper 95% Mean	1.009392	0.9413131	0.9235293	1.0070098	1.1717765	1.1057922	1.5943627	1.6008747
Lower 95% Mean	0.990608	0.9216278	0.9041438	0.9780902	1.1338409	1.0544608	1.5288034	1.5221859
N	946	1074	1024	1634	2220	1248	1583	989

Supplementary Figure 11: Outlier box-plots of time-resolved Akt phosphorylation in MDA-MB-231 cells after stimulation with 50 ng mL^{-1} PDGF-BB for 0-7 min. The box represents the inter-quartile range (IQR, 3rd quartile minus the 1st quartile) and the errors bars (whiskers) represent + or - 1.5×IQR from the 3rd or 1st quartile, respectively. Cells with responses greater than 75.0% quartile plus 1.5×IQR were considered hyperactive responders.

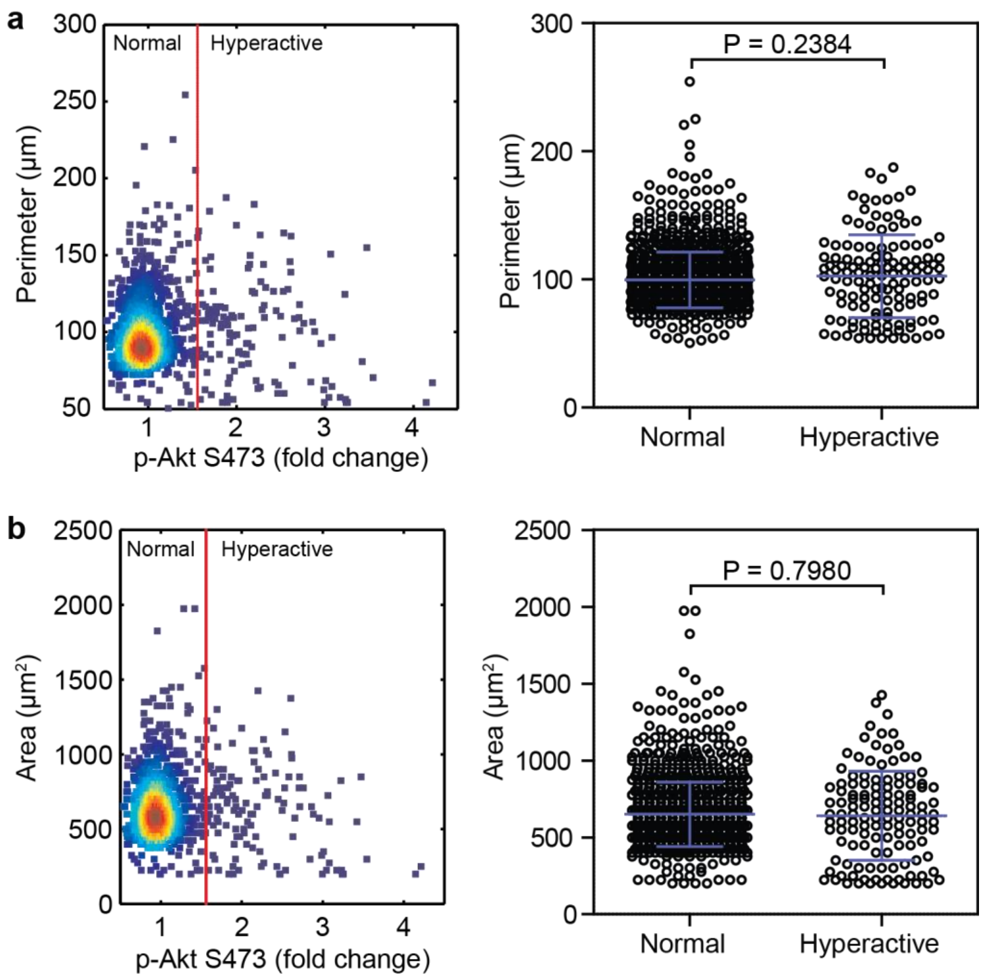

Supplementary Figure 12: 2D profile heat maps (left; red = high population and blue = low population) and single-cell scatter plots (right) comparing normal and hyperactive populations of MDA-MB-231 by area (**a**) and perimeter (**b**). The red vertical line represents the threshold for hyperactive activation (3rd quartile + 1.5×IQR). Error bars are population mean +/- S.D. and scatter plots were generated from at least 3 different day replicates (representing a total of 946-2219 cells per condition). Data were considered statistically significant at $p<0.05$.

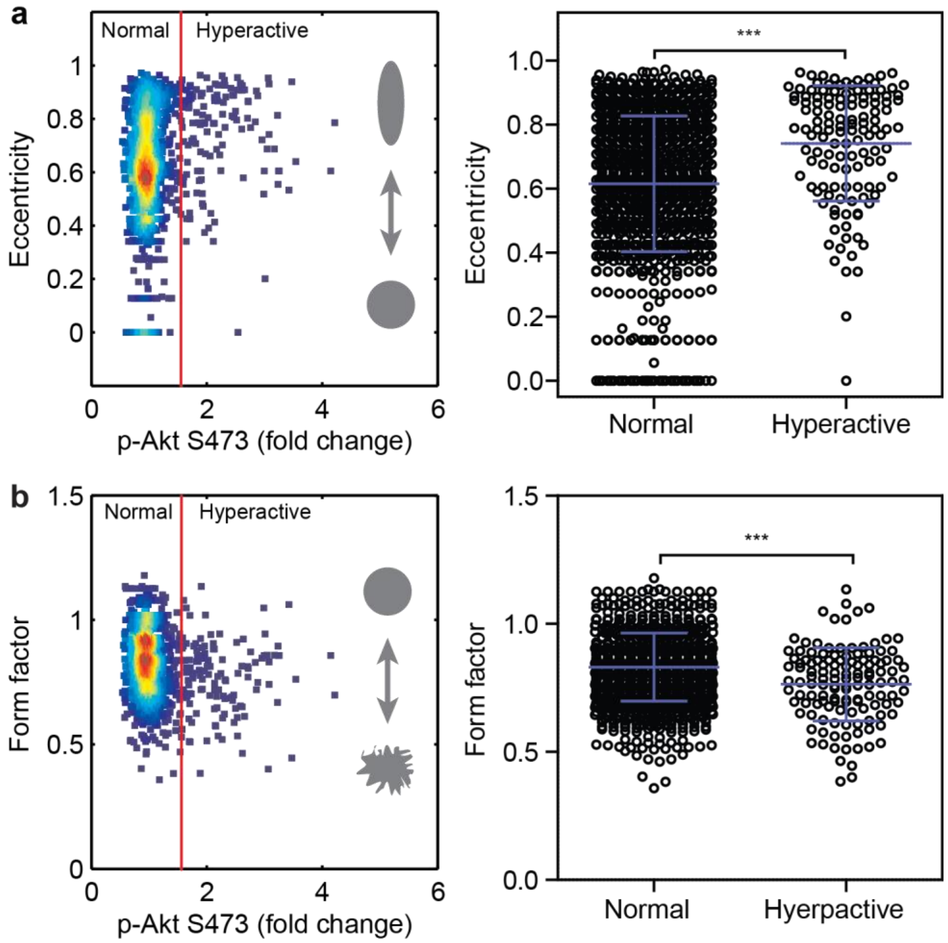

Supplementary Figure 13: 2D profile heat maps (left; red = high population and blue = low population) and single-cell scatter plots (right) comparing normal and hyperactive populations of MDA-MB-231 by eccentricity (**a**) and form factor (**b**). The red vertical line represents the threshold for hyperactive activation (3rd quartile + 1.5×IQR). The grey cartoons indicate the trend in shape-change. Error bars are population mean +/- S.D. and scatter plots were generated from at least 3 different day replicates (representing a total of 946-2219 cells per condition); ***$p<0.001$. Data were considered statistically significant at $p<0.05$.

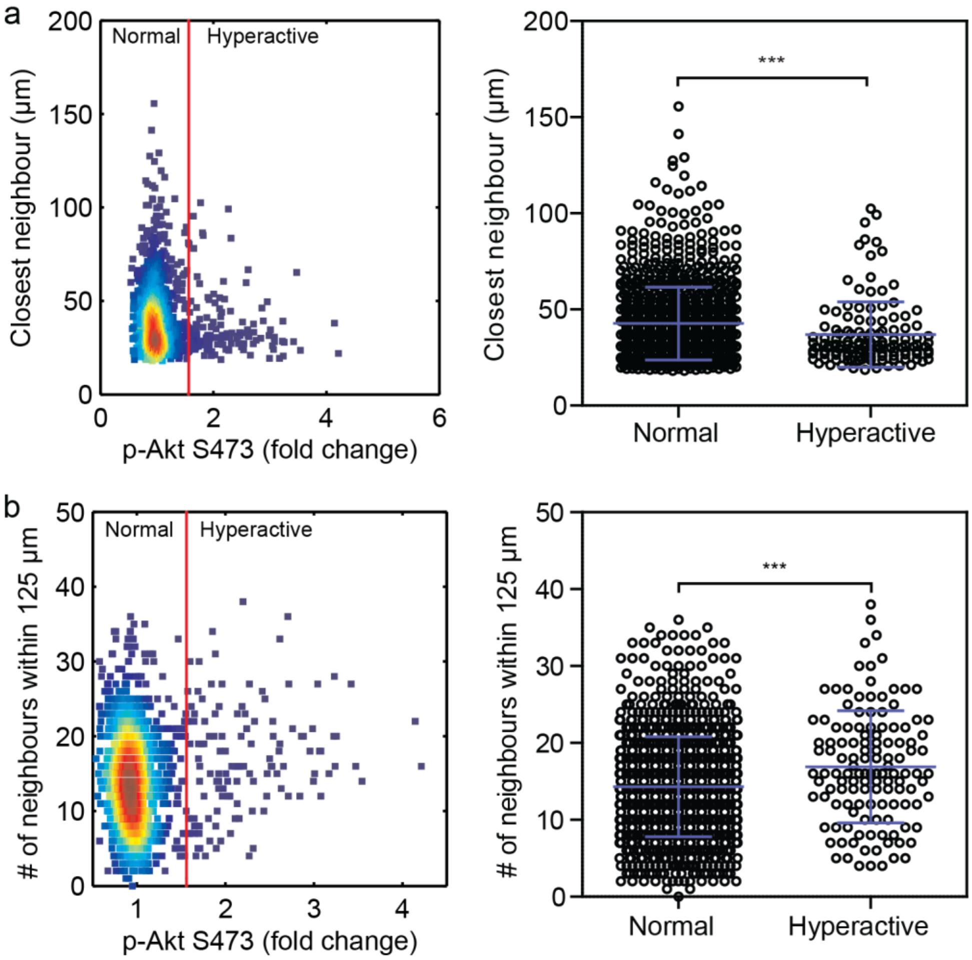

Supplementary Figure 14: 2D profile heat maps (left; red = high population and blue = low population) and single-cell scatter plots (right) comparing normal and hyperactive populations of MDA-MB-231 by distance of closest neighbour (**a**), and number of neighbours within 125 μm (**b**). The red vertical line represents the threshold for hyperactive activation (3rd quartile + 1.5×IQR). Error bars are population mean +/- S.D. and scatter plots were generated from at least 3 different day replicates (representing a total of 946-2219 cells per condition); ***$p<0.001$. Data were considered statistically significant at $p<0.05$.

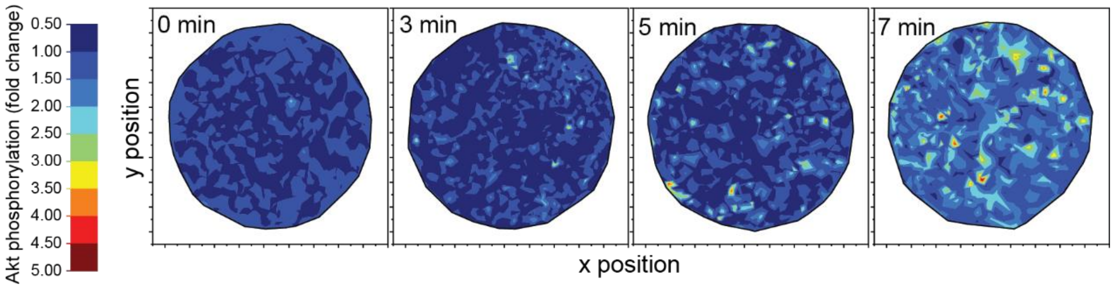

Supplementary Figure 15: Contour plots of Akt phosphorylation in MDA-MB-231 cells as a function of position in a virtual microwell after stimulation with 50 ng mL^{-1} PDGF-BB for 0, 3, 5, or 7 min. Each tick in the axis represents 100 μm in the virtual microwell. Contour plots were generated from at least 3 different day replicates, representing a total of 946-1634 cells per condition.

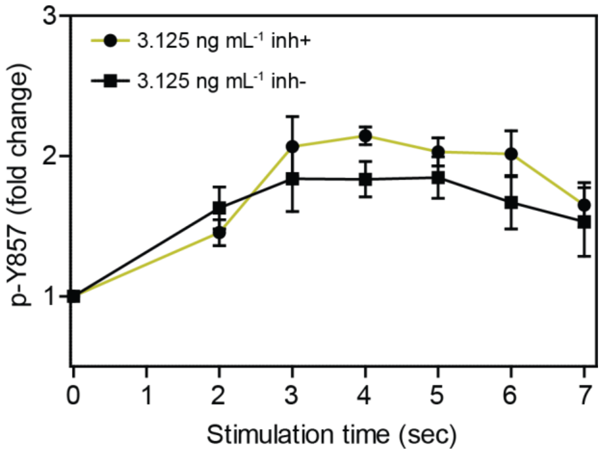

Supplementary Figure 16: Plot showing PDGFR Y857 phosphorylation in NIH-3T3 cells stimulated for 0-7 min with 3.125 ng mL^{-1} PDGF-BB in the presence (inh+, circles) or absence (inh-, squares) of phosphatase inhibitor sodium orthovanadate (10 mM). Data in plot are average fold changes with respect to zero (no stimulation) +/- S.E.M. from at least 4-5 virtual microwells performed on different days, representing 592-1280 cells per condition. Inhibitor did not cause a significant difference (p = 0.1163, two-way ANOVA) in cell signaling.

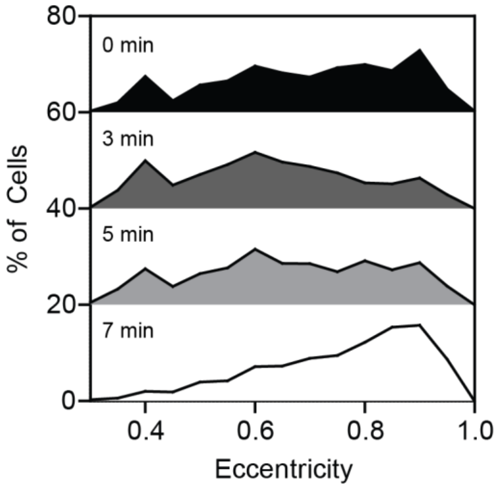

Supplementary Figure 17: Histograms of eccentricity in MDA-MB-231 cells after stimulation with 50 ng mL^{-1} PDGF-BB for 0, 3, 5, or 7 min. Histogram distributions were generated from at least 3 different day replicates (representing a total of 946-1634 cells per condition), and were offset vertically for comparison.

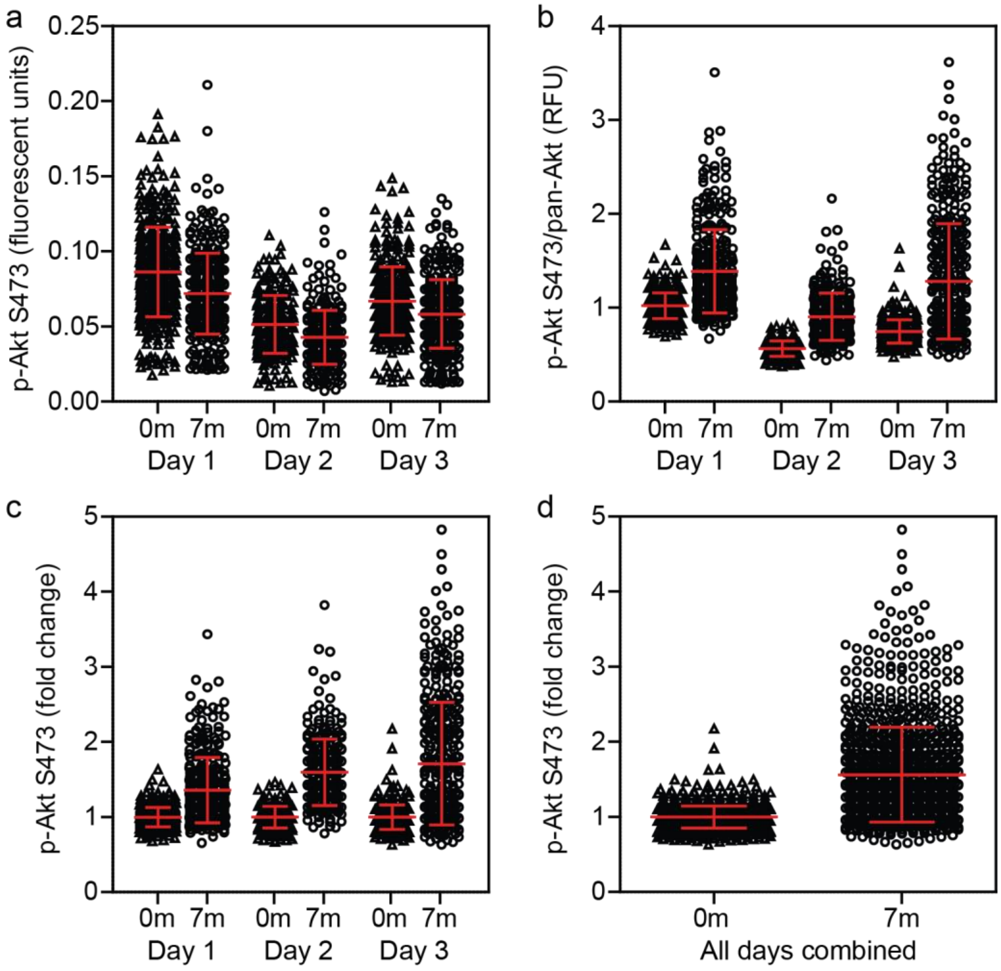

Supplementary Figure 18: Example data analysis for Akt phosphorylation of MDA-MB-231 cells after 0 or 7 min stimulation with 50 ng mL^{-1} PDGF-BB. (**a**) Different day single-cell scatter plots of fluorescent intensity from Cy3 channel (p-Akt S473). (**b**) Different day single-cell scatter plots of fluorescent intensity from Cy3 channel relative to Cy5 channel (p-Akt S473/pan-Akt). (**c**) Different day single-cell scatter plots of p-Akt S473/pan-Akt normalized to average of non-stimulated control. (**d**) Combined single-cell scatter plots from different days. Error bars on different day scatter plots represent population mean +/- S.D. from 217-391 cells. Errors bars on combined scatter plots represent population mean +/- S.D. from 946-989 cells.

Supplementary Note 1: Estimation of shear stress on cells

The wall shear stress on cells adhered in a virtual microwell was estimated by equation S1, adapted from Nauman et al. *Annals of Biomedical Engineering* **27**, 194-199 (1999):

$$\tau_{wall} = \frac{\mu 6\overline{V}}{h} \qquad \text{(S1)}$$

where τ_{wall} is wall shear stress, μ is viscosity coefficient of the liquid, $\overline{V}$ is the average velocity of the droplet, and h is the gap height of the device. Assuming DI water at 25°C is used (μ = 0.894×10^{-3} Pa s), a droplet takes ~3 seconds to perform passive dispensing (from merge to split) on a 1.75 mm diameter spot ($\overline{V}$ = 1.75 mm s^{-1}), and gap height of 180 µm, then τ_{wall} = ~0.52 dynes cm^{-2}.
